# Polish adaptation and validation of the sustainable career scale

**DOI:** 10.1371/journal.pone.0357496

**Published:** 2026-09-08

**Authors:** Jaroslaw Grobelny, Angela Russo, Mateusz Klakus, Andrea Zammitti

**Affiliations:** 1 Faculty of Psychology and Cognitive Science, Adam Mickiewicz University, Poznań, Poland; 2 Department of Human and Social Sciences, University Mercatorum of Rome, Rome, Italy; 3 Department of Educational Sciences, University of Catania, Catania, Italy; Universiti Pertahanan Nasional Malaysia, MALAYSIA

## Abstract

The evolving global labor market highlights the significance of sustainable careers, which balance individual well-being, productivity, and social empowerment. This study aimed to adapt the Sustainable Career Scale for the Polish context, addressing the need for culturally transferable models to support evolving career paths. Understanding these cultural influences is crucial for advancing global career research. The study followed a rigorous, multi-stage process involving mixed methods. Focus group interviews were conducted to assess cultural relevance, item clarity, and face validity. The pilot study tested reliability and internal consistency using exploratory factor analysis. Study 1 utilized confirmatory factor analysis to verify the four-factor structure and evaluate measurement invariance across gender and age groups. Study 2 assessed lagged associations with well-being outcomes and test-retest reliability through longitudinal data collection. The Polish adaptation of the SCS maintained its four-factor structure (happiness, health, productivity, and social empowerment) and demonstrated high internal consistency and temporal stability, along with criterion and discriminant validity evidence and lagged associations with well-being outcomes, with some limitations for the Health dimension. The scale showed measurement invariance across gender and tested age groups. This study provides preliminary evidence supporting the Polish adaptation of the SCS in the present samples, confirming the distinctiveness of its four dimensions. The findings support the applicability of the sustainable careers model in the Polish context and underscore the significance of sustainable career strategies.

## Introduction

The contemporary labor market is undergoing profound changes driven by rapid technological advancements, globalization, and socioeconomic shifts [1]. These changes transform traditional career paths and pose significant employee risks and challenges. The rise of automation and artificial intelligence is reshaping job roles, leading to job displacement and requiring workers to continually update their skills to remain competitive [2].

The rise of gig and freelance work has increased job insecurity, contributing to burnout and turnover intentions, trends exacerbated by the COVID-19 pandemic through the expansion of remote work [3,4]. These shifts necessitate a re-evaluation of career management strategies to ensure long-term career sustainability. As such, the concept of a sustainable career has gained recognition as crucial, building on a long history of career-focused research in work psychology [5]. Achieving a sustainable career has become more challenging due to labor market volatility, driven by technological change and economic uncertainty, demanding greater proactivity and resilience [6]. Therefore, studying how individuals and organizations can create environments that support sustainable career trajectories is crucial, ultimately leading to more satisfied and productive workforces. Understanding the mechanisms of career sustainability is essential today, as it can guide policies and practices that enhance long-term employee well-being and organizational success.

A career is a sequence of work experiences, education, and training across organizations and contexts, shaping an individual’s professional life [7,8]. It is a dynamic process evolving over time [9], dependent on context [10], and requires continuous negotiation with institutions and society, forming both personal and social trajectories [11]. Currently, work psychology views careers as multidimensional, non-linear processes shaped by developmental stages, life roles, individual meaning-making, and cognitive, social, and experiential factors. [12–14]. Career trajectories have become less stable due to demographic, economic, social, and technological shifts [4,15–17], requiring adaptive strategies in volatile markets [18,19]. These changes led to the development of new paradigms such as boundaryless, protean, and sustainable career models [15,20–22], to reflect a shift from traditional, linear career paths to more systemic, dynamic, and individual-driven career development. Notably, career sustainability has become a crucial framework, balancing individual aspirations with societal demands while integrating individual, contextual, and temporal dimensions of career development [8,23].

Newman [21] and De Vos et al. [7] present two key models of sustainable careers. Newman outlines three core elements: renewability, supporting extended working lives and life balance; flexibility and adaptability, enhancing resilience; and ego integrity. Chin et al. [24] build on this with a four-dimensional scale: renewability, flexibility, balance, and resourcefulness. De Vos et al. view sustainable careers as evolving work experiences shaped by various social contexts and life stages. These include temporal dynamics, emphasizing both short-term disruptions and long-term shifts [6,25]; context, highlighting interdependent personal and professional roles within organizations, social settings, and family [22,26]; and the personal dimension, centering on the individual as the key agent who integrates personal and contextual resources to adapt and achieve career fit [27,28].

According to De Vos et al. [7], a sustainable career enables the development of health (e.g., both mental and physical, resulting from demands-resources and work-life balances), happiness (e.g., career satisfaction and career success), and productivity (e.g., perceived good performance and employability across different life stages). After careful theoretical considerations and a series of mixed-method studies, Russo et al. [8,29] expanded their model of sustainable careers to include a fourth dimension: social empowerment. This addition emphasizes the significance of ethical contributions to society. As such, this model identifies happiness, health, productivity, and social empowerment as critical indicators of a sustainable career.

This study seeks to develop and validate the Polish version of the Sustainable Career Scale (SCS [29]), a psychological instrument crafted to assess career sustainability as conceptualized by Russo et al. [8]. Adhering to rigorous standards [30], the adaptation process involved a mixed-methods approach, encompassing a pilot study and two independent primary studies to evaluate this novel test’s psychometric attributes rigorously. The first study, utilizing an observational design, examined the scale’s reliability, factor structure, and measurement invariance. The second study, involving follow-up measurement, aimed to evaluate the scale’s reliability and validity, specifically criterion and discriminant validity, as well as its lagged associations with well-being outcomes. As such, the research makes two substantial contributions to the field of career psychology. Firstly, it introduces a robust instrument for assessing a pivotal aspect of individual careers, potentially enhancing career path development and integrating the United Nations Sustainable Development Goals (SDGs) into the workplace. Secondly, it offers empirical support for Russo et al.’s innovative theoretical model of career sustainability by adapting and validating the scale within a new cultural context. As the original scale has already been validated for factor structure, reliability, validity, predictive validity, and gender invariance [29], the present contribution lies in extending this evidence to the Polish cultural context, providing an initial indication that the model’s core dimensions are recognizable beyond the setting in which it was originally developed.

## SCS cultural adaptation

This research project sought to adapt an Italian-developed scale for assessing career sustainability to the Polish cultural context. IT is essential to deepen the understanding of how cultural factors shape career perceptions and expectations, as cultural values such as future orientation, collectivism, and power distance significantly influence career success definitions [31]. This adaptation will help advance career theory by ensuring that assessments are sensitive to the diverse cultural contexts within and across nations, thereby improving both theoretical models and practical applications [32,33].

The SCS [29] is a multidimensional scale designed to assess the subjective sense of career sustainability among workers. Rooted in the model of Russo et al. [8], the SCS was developed through a five-step process involving multiple studies. The scale’s latent structure, consisting of four distinct factors (happiness, health, productivity, and social empowerment) converging into a higher-order factor of sustainable career, has been consistently confirmed across different samples and validated for gender invariance. Moreover, the SCS exhibits excellent concurrent and discriminant validity, demonstrating positive correlations with work engagement, work meaning, work-family balance, decent work, employability, and job satisfaction while negatively correlating with burnout. Furthermore, the SCS has been found to predict hedonic, eudaimonic, and social well-being, highlighting its relevance for understanding the broader impact of career sustainability on individuals’ overall quality of life.

### Initial steps and translation process

The adaptation of the SCS followed the International Test Committee guidelines [30]. Initially, necessary permissions were secured from the copyright holders, and the applicability of the career sustainability construct within Polish culture was evaluated. The construct was deemed suitable for adaptation; however, some concerns regarding its structure also prompted the decision to employ a qualitative approach. Subsequently, the translation process commenced. Two work psychologists, proficient in English, independently performed forward translations. Additionally, a generative AI model (GPT-3.5) was utilized as a supportive tool for translation, specifically instructed to account for cultural nuances in the translation task. The two psychologists’ translations were then compared during a joint session. Both were additionally provided with the GPT-3.5 translation, which they evaluated by comparing its differences against their own versions. Through discussion, they integrated their two translations into a single version, resolving any discrepancies collectively, with the GPT-3.5 version available to them only as supplementary, informative material rather than as an authoritative source.. An integrated version of the translation was then reviewed by a third work psychologist, previously uninvolved in the project, who performed a backward translation. This version was also scrutinized for discrepancies addressed through collective discussion among the authors. Further evaluation was conducted by an expert in Polish linguistics (PhD holder), who assessed the language for correctness and naturalness. In the final stage, discussions were held with the original authors of the scale to ensure fidelity to the intended meanings of the items. This refined version underwent a qualitative evaluation to gauge the test-takers’ perspectives and their understanding of the items.

### Test takers’ perspective and cultural transferability

Focus group interviews (FGIs) were conducted to evaluate the applicability of the four-factor sustainable career model within the Polish cultural context. A qualitative approach was deemed appropriate at this stage, as FGIs in psychometric research can enhance the tool’s reception, comprehension, and face validity [34]. The primary objective was to assess participants’ understanding of specific test items and identify potential barriers to effective implementation. A detailed summary of item-level comprehension data and revision decisions is provided in S1 File.

#### Participants and groups.

Participants were recruited through announcements shared on local social media communities. Two homogeneous groups were formed according to educational background and occupational type. Based on the applications, participants were invited to one of two groups: (1) blue-collar workers (e.g., retail salesperson; *n* = 3, all women, age range: 28–33); (2) white-collar workers with higher education (e.g., school pedagogue; *n* = 5, 4 women, age range: 23–31). In total, the sample comprised eight participants, of whom seven were women. Participants were compensated with a prepaid card (PLN 150).

#### Procedure.

Focus groups were conducted in a dedicated room, with two trained assistants managing technical support and interviewing roles. The FGI scenario was developed according to Rupp’s [34] guidelines and involved three phases: (1) an integration phase, in which participants were introduced to each other and to the subject of the interview through career-related association tasks; (2) an individual work phase, in which each participant completed the SCS and then evaluated each item for clarity and ease of response (“Was question X clear?”, “Did you know how to answer it?”), and provided general written feedback; and (3) a group discussion phase, covering general reflections and item-by-item discussion of the questionnaire.

#### Data analysis.

Following the interviews, each session was transcribed, and participants’ responses to individual items and the overall questionnaire were digitized. Two researchers independently reviewed the statements and categorized them into two themes: item wording and construct validity.

The item wording theme focused on comprehension and interpretation of specific test items. Participants in the white-collar group reported difficulty responding to Happiness subscale items (items 1–3) due to conceptualizing career as a finite, already-completed sequence rather than an ongoing process. Comprehension difficulties with the Social Impact subscale items were evident in both groups. Participants’ statements indicated (1) high content convergence among the three Social Impact items, making them difficult to distinguish, and (2) perceived inadequacy of the subscale content for their occupational context.

The construct validity theme encompassed statements regarding expected questionnaire content and differing lay understandings of sustainable career. References to constructs such as work-life balance and work importance were evident across both groups, consistent with findings reported by Russo et al. [8]. The differential nature of the Social Impact subscale was also highlighted, suggesting that its relevance may vary substantially depending on occupational role and sector.

## Pilot study

### Method

The pilot study was exploratory in nature; consequently, no explicit hypotheses were formulated. Nonetheless, the study aimed to evaluate the reliability and structure of the questionnaire results to determine whether the tool is suitable for more rigorous subsequent testing. Data for the pilot study were collected between March 5^th^ and April 2^nd^, 2024. The procedures used in this study adhere to the tenets of the Declaration of Helsinki. Informed consent was obtained via online form from all participants before they participated in the Pilot and each subsequent study. The consent process was conducted via an online testing platform, ensuring that all individuals were fully informed about the nature and purpose of the research. Participants provided explicit agreement to take part in the study and consented to the use of their data for publication. Prospective ethical review was not required for this study, which involved minimal-risk, anonymous survey research with consenting adults and no sensitive or potentially harmful content. The project was subsequently submitted for retrospective review and received a positive opinion from the Ethics Committee for Research Projects of Adam Mickiewicz University in Poznań (Opinion no. 07/03/2026, issued 17 March 2026); the application covered the full research programme and stated that data collection had already been concluded.

#### Participant and sampling.

The inclusion criteria for the pilot study, as well as for the subsequent main studies, required participants to be actively employed. To safeguard the validity of the results, specific exclusion criteria were implemented: individuals were excluded if they were retired, under 18, or had less than three months of work experience. Additionally, migrants without Polish citizenship were excluded to prevent potential variability in career evaluation outcomes, as their inclusion could significantly skew the results. The pilot study aimed to enroll a minimum of 100 individuals. The sample size was determined based on the minimum required ratio of participants to the number of test items [35,36].

Of the 126 participants who commenced the study, 108 completed it, and 100 met the eligibility criteria. Of these, 80% were women, 18% were men, and the remainder did not specify their gender. The average age of participants was 34.7 years (*SD* = 10.9), with an average of 12.4 years of professional experience (*SD* = 9.9). The sample composition was predominantly office workers, accounting for 74%, followed by manual workers at 15% and professional caregivers at 11%. The majority held full-time work contracts (67%), while others were employed under civil contracts (18%) or operated as sole proprietors (10%). Regarding industry, 88% of participants worked in service sectors, with 72% possessing higher education and 27% secondary education.

Given the exploratory nature of the study, convenience sampling was employed. The study description was disseminated through social media groups associated with major Polish cities. Interested participants accessed the study via a provided link, which redirected them to an online testing platform hosted on the university server. Here, they encountered an introductory page that outlined the study details and their rights. Participants advanced to the subsequent sections after providing informed consent and passing the eligibility verification.

#### Measurements.

*Career sustainability* was assessed using the Polish version of the SCS, which consists of 12 items divided into four scales. Each item was structured as a statement, and participants were required to respond using a 7-point Likert scale. The three health items (items 4–6), which are negatively worded, were reverse-scored prior to the calculation of scale totals and all substantive analyses, so that higher scores indicate a higher level of the respective dimension. Item-level descriptive statistics reported in Table 1 are presented on the original, non-reversed item scale. The terms “career” and “work” were both used in the items, with “career” being defined in the instruction as the overall work experience accumulated throughout one’s life, and “work” referring to current job activities. In addition to the SCS, only demographic data were collected in the pilot study.

**Table 1:** Descriptive Statistics and Corresponding Factors Loadings from Pilot and Study 1.

Item	*M* [*LL*, *UL* 95% CI]	Kurt	Skew	*W*	Δ*α*^a^		loadings^a^
					general^b^	dimension^c^	
1) Moja kariera pozwala mi być ogółem zadowolonym(-ą) z życia.	4.77 [4.64, 4.9]	−0.59	−0.03	.92***	−.02|−.03	−.06|−.06	.96|.90
2) Jestem zadowolony(-a) z tego, jak rozwija się moja kariera	4.57 [4.44, 4.71]	−0.46	−0.4	.93***	−.02|−.03	−.04|−.05	.86|.80
3) Praca, którą wykonuję sprawia, że czuję się spełniony(-a) w życiu.	4.23 [4.08, 4.37]	−0.23	−0.8	.94***	−.03|−.03	0|−.02	.68|.89
4) Często budzę się w nocy myśląc o mojej pracy.	2.59 [2.45, 2.73]	0.97	0.16	.86***	.01|.01	.01|−.02	.66|.60
5) Obawiam się, że poziom stresu w mojej pracy wywoła u mnie jakieś problemy zdrowotne.	3.43 [3.27, 3.59]	0.35	−1.02	.92***	.01|0	−.09|−.22	.88|.86
6) Moja praca sprawia, że często jestem tak zmęczony(-a), że nie mogę się na niczym skupić.	3.52 [3.37, 3.67]	0.37	−0.84	.93***	0|0	−.11|−.15	.84|.76
7) Myślę, że jestem kompetentny(-a) w tym, co robię w pracy.	5.78 [5.69, 5.87]	−0.98	1.84	.86***	0|−.01	−.04|−.06	.96|.77
8) Myślę, że jestem przygotowany(-a) do wykonywania mojej pracy.	5.72 [5.63, 5.81]	−0.78	0.76	.87***	0|0	−.03|−.07	.91|.75
9) Moim zdaniem jestem dobry(-a) w tym, co robię zawodowo.	5.69 [5.59, 5.78]	−0.65	0.5	.88***	0|−.01	0|−.02	.86|.94
10) Poprzez moją karierę mogę przyczynić się do budowania lepszego społeczeństwa.	4.56 [4.41, 4.71]	−0.35	−0.65	.94***	−.02|−.02	0|−.01	.78|.89
11) Czuję, że dzięki mojej karierze pomagam ludziom żyć lepiej.	4.48 [4.33, 4.63]	−0.31	−0.79	.94***	−.02|−.02	−.04|−.06	.97|.95
12) Moja kariera w istotny sposób przyczynia się do szczęścia społeczności i innych ludzi.	4.36 [4.21, 4.51]	−0.23	−0.75	.94***	−.02|−.02	−.02|−.02	.94|.86

*Note*. *N* = 100 (Pilot), 497 (Study 1). *M* = mean; *LL* = lower limit; *UL* = upper limit; 95% CI = 95% confidence interval; *Kurt* = kurtosis; *Skew* = skewness; *W* = Shapiro-Wilk test results; Δ*α* = change in Cronbach’s alpha if the item were removed; negative values indicate that removing the item would reduce scale reliability; loadings = factor loadings for the corresponding factor per item, obtained from an exploratory factor analysis for the Pilot and from the confirmatory factor analysis of the four-factor correlated model for Study 1. Descriptive statistics for items 4–6 (Health) are reported prior to reverse scoring.

a values are presented for the Pilot and Study 1, separated by vertical bar; ^b^values appropriate for calculating general SCS score; ^c^values appropriate for calculating dimensional scores.

***p < .001.

#### Data analysis approach.

Due to the online testing format and the inclusion of mandatory questions, there was no missing data in the survey. No additional steps were taken to ensure data quality in the pilot study. The analysis began by calculating internal stability coefficients, including Cronbach’s alphas and McDonald’s omegas. In the latter, four factors and the minimum residuals method were utilized for calculation. To proceed, both the scale and its subscales were required to meet a minimum threshold of.60 for all these metrics. The analytical process culminated in an EFA, which presumed a four-factor structure. The minimum residuals method was utilized for factor extraction, coupled with an oblimin rotation to allow for correlations between factors. To advance the development of the SCS, each item was expected to achieve a minimum factor loading of 0.60 on its corresponding factor. The analysis was conducted using the *R* language [37], employing the *psych* package [38].

### Results

First, a reliability analysis was conducted, yielding exceptionally high coefficients: Cronbach’s alpha for the entire scale was.87, and McDonald’s omega was.96. Internal stability coefficients for the subscales were also very high, with only one falling below.90 (.93,.84,.93, and.95, respectively). Notably, in the subscale analysis, no item removal resulted in an increase in the alpha level, as shown in Table 1. When analyzing the entire scale, two items exhibited only a negligible increase in internal stability when dropped (by.01).

Next, an EFA was conducted. Initial tests verified the data’s suitability for factor analysis, with Bartlett’s Test of Sphericity (*χ*^2^ = 1053.87; *df* = 66; *p* < .001) and the Kaiser-Meyer-Olkin measure (.82) both supporting this. The actual EFA yielded satisfactory results. As indicated in Table 1, the majority of loadings met the criteria for an excellent value (>.71 [39]), and the rest were very good (>.63). Full details of the EFA, including the complete pattern matrix, communalities, eigenvalues, parallel analysis, and inter-factor correlations, are provided in S1 File. This analysis enabled us to proceed with further validation of the Polish version of the SCS (available at the Open Science Framework repository at https://osf.io/2n4r5/files/32va5, which initially indicated satisfactory psychometric properties.

## Study 1: Observational study

The primary aim of Study 1 was to evaluate the psychometric properties of a culturally adapted scale. To this end, a series of hypotheses were formulated.

*H1. The factor structure of the SCS is best represented by a hierarchical model, wherein a single general factor underpins four second-order factors.*
*H2. The SCS exhibits measurement invariance across groups differentiated by participants’ (a) gender and (b) age.*
*H3. The SCS is a reliable measurement of career sustainability in the Polish context.*

The validation of Hypothesis 1 would support the scale’s validity, consistent with Russo et al.’s [8,29] theory that posits a single general factor underpinning four second-order factors within a sustainable career structure. Verifying Hypothesis 2 would provide additional support for the scale’s reliability, demonstrating the consistency of latent constructs across different demographic groups. Specifically, gender groups were selected to replicate Russo et al.’s [29] original findings, while comparisons across age groups were intended to explore the potential effects of aging on career sustainability [40], an aspect not previously examined. Hypothesis 3 was established to confirm that the adapted scale maintains satisfactory internal consistency, thereby affirming its reliability.

### Method

#### Participant and sampling.

The same eligibility criteria used previously were applied to Study 1. The target sample size was set at 350 individuals. This number was determined using sample size calculation rules for confirmatory factor analysis, the primary statistical method employed in this study. Based on the expected moderate effect size derived from Russo et al. [29], a target statistical power of.95, an alpha level of.05, five latent variables, and twelve observed variables, the minimum sample size required to analyze the model structure was calculated to be 308, with a recommended size of 341 individuals [41]. Considering potential data loss due to procedural errors and following Comrey and Lee’s [39] guidelines for a “good” sample size, the recruitment goal was established at 350 participants. A stopping rule was implemented to cease active recruitment upon reaching the minimum sample size and conclude the study one week later.

Of the 654 individuals who began the survey, 561 completed it. From this group, 52 participants were excluded based on eligibility criteria, and an additional 12 were excluded following attention checks, yielding a final sample of 497 participants. The critical *𝜒*^2^ values were 65.17 and 67.50, based on sensitivity analyses using 1 − *β* and *α* levels of.95 and.05, and degrees of freedom of 48 and 50, respectively.

The participant group consisted of 70.8% women and 26.4% men, with the remaining respondents not disclosing their gender. The average age was 30.4 years (*SD* = 8.8), and they had an average of 8.7 years of professional experience (*SD* = 7.8). Office workers made up 67% of the sample, manual workers 23.7%, and 9.3% engaged in both types of work. A majority of the sample, 55.7%, were employed under employment contracts, while 30.6% worked under civil contracts, and 7.4% were sole proprietors. The vast majority, 88.9%, worked in the service sector, and 71.2% had higher education. Surveyed employees worked an average of 34.8 hours per week (*SD* = 13.4), and 11.1% of them worked remotely (*SD* = 14.6).

#### Data collection procedure and measurements.

The procedure the respondents underwent, including informed consent and the ethical arrangements described above, was identical to that of the Pilot study, except for introducing two attention checks. Study 1 employed the exact measurements and informed consent collection (via online form) as the previous study. Data for Study 1 were collected between May 6^th^ and June 28^th^, 2024.

#### Data analysis approach.

Due to the online testing format and the inclusion of mandatory questions, the survey did not have any missing data. Two attention checks were integrated into the survey to enhance data quality further. The first was embedded among the SCS items and required participants to select a specific response. The second check was positioned at the end of the study and asked participants to self-report whether their responses were provided reliably and attentively.

This study computed descriptive statistics at both the item and scale levels, incorporating means with 95% confidence intervals, Shapiro-Wilk tests for assessing distribution normality, and measures of kurtosis and skewness. The analysis proceeded with an evaluation of the square root of average variance extracted (AVE) within each subscale, compared against the correlations among factors to preliminarily assess the convergence of the subscales. It was anticipated that the square root of the AVE for each factor would surpass its correlations with other factors.

CFA was performed using the R package *lavaan* [42], with models estimated by robust maximum likelihood (MLR). The theoretical model—a hierarchical structure with one general factor and four first-order factors—was evaluated against a series of competing models, as delineated in the literature and previously analyzed by Russo et al. [29]. Given prior debates about the social factor, we also considered a three-first-order factors model, integrating Social Empowerment into Productivity. These models were assessed using a comprehensive array of fit and error indices, including *χ*^2^, GFI, AGFI, TLI, PGFI, RMSEA, and SRMR, to cover a spectrum of fit measures that reflect absolute, relative, and parsimonious fit, as well as noncentrality and absolute residual-based indices. To support Hypothesis 1, the hierarchical four-factor model needed to demonstrate superiority.

Measurement invariance was tested across (a) gender and (b) age to verify that the scale measures the constructs equivalently across groups. A multigroup approach was used, fitting the four-factor model under progressively restrictive constraints (configural, metric, scalar, and residual). Age groups were defined generationally following Robinson [43]: Generation Z (aged 18–28), Generation Y (29–42), and Generation X (43–58). Support for Hypothesis 2 required no substantial deterioration in fit across the constraint levels.

Finally, reliability analyses were re-conducted to confirm the internal consistency of the scale using Cronbach’s alpha and McDonald’s omega. With a larger sample size and improved sampling procedures, a threshold of.70 was expected for these reliability measures to confirm Hypothesis 3.

### Results

Table 1 displays descriptive statistics derived from Study 1. Although all items deviated from a normal distribution, they exhibited reasonable values for skewness and kurtosis. Only four items showed moderate skewness, while the remainder were symmetrical. Kurtosis values were even more favorable; only one item approached a leptokurtic distribution, but overall, all 12 items suggested a mesokurtic distribution. Additionally, the square roots of AVE ranged from.62 (for Health) to.92 (for Social Empowerment), and the correlations among factors did not exceed.50. While each value exceeded the corresponding inter-factor correlations, the value for Health was comparatively low, indicating limited convergence among its items. Only two factors, Health and Productivity, were negatively correlated, which is theoretically justified. Overall, these results, outlined in Table 2, provide initial support for the discriminant validity of the SCS in terms of its subscales.

**Table 2:** Square Root of Average Variance Extracted and Correlations between Factors.

Factor	1.	2.	3.	4.
1. Happiness	(.88)			
2. Health	.29***	(.62)		
3. Productivity	.27***	−.18**	(.83)	
4. Social Empowerment	.50***	.07*	.17**	(.92)

*Note*. *N* = 497. Diagonals present square root AVE values for factors

*p < .05. **p < .01. ***p < .001.

Subsequently, competing models were analyzed through CFA, with the results detailed in Table 3. Considering all metrics, only the four-factor models achieved a fit that met the recognized criteria for acceptability [44,45], and none of the models competitive with the theoretical constructs (i.e., with single or three factors specified) achieved satisfactory relative fit (>.90) or error metrics (≤.08).

**Table 3:** Comparison of Fit Indices for Different Model Structures and Measurement Invariance Analysis.

Model	n	*χ* ^2^	df	GFI	AGFI	TLI	PGFI	RMSEA	SRMR	AIC	BIC
single factor	497	2250.47***	54	0.55	0.35	0.27	0.38	0.29	0.20	20006.09	20107.05
three orthogonal factors	497	978.21***	54	0.73	0.61	0.69	0.51	0.19	0.16	18733.83	18834.79
three related factors	497	950.46***	51	0.74	0.60	0.68	0.48	0.19	0.14	18712.08	18825.65
three hierarchical factors	497	952.51***	51	0.74	0.60	0.68	0.48	0.19	0.14	18714.13	18827.71
four orthogonal factors	497	381.25***	54	0.89	0.84	0.89	0.62	0.11	0.18	18136.87	18237.83
four related factors	497	170.98***	48	0.94	0.91	0.95	0.58	0.07	0.05	17938.60	18064.8
four hierarchical factors	497	202.48***	50	0.93	0.90	0.95	0.60	0.08	0.08	17966.10	18083.89
**Multigroup analysis: gender**	***χ*2**	**df**	**CFI**	**RMSEA**	**SRMR**	**Δχ²**	**Δdf**	**p**	**ΔCFI**	**ΔRMSEA**	**ΔSRMR**
configural	226	96	0.954	0.075	0.055						
metric	226	104	0.957	0.07	0.056	1.5	8	0.993	0.003	−0.005	0.001
scalar	237	112	0.956	0.068	0.057	10.4	8	0.24	−0.001	−0.002	0.001
residual	233	124	0.962	0.06	0.057	4.47	12	0.973	0.006	−0.008	0
Multigroup analysis: age	***χ*2**	**df**	**CFI**	**RMSEA**	**SRMR**	**Δχ²**	**Δdf**	**p**	**ΔCFI**	**ΔRMSEA**	**ΔSRMR**
configural	182	96	0.965	0.064	0.055						
metric	198	104	0.961	0.065	0.061	17	8	0.031	−0.004	0.001	0.006
scalar	222	112	0.954	0.068	0.063	24.6	8	0.002	−0.007	0.003	0.002
residual	219	124	0.961	0.06	0.064	6.31	12	0.9	0.007	−0.008	0.001

*Note*. *χ*² = chi-square goodness-of-fit test; df = degrees of freedom; GFI = goodness-of-fit index; AGFI = adjusted goodness-of-fit index; TLI = Tucker-Lewis index; PGFI = parsimony goodness-of-fit index; RMSEA = root mean square error of approximation; SRMR = standardized root mean square residual; AIC = Akaike information criterion; BIC = Bayesian information criterion.

***p < .001.

The hierarchical four-factor model exhibited a superior parsimonious fit index, which is more effective in evaluating complete models. Conversely, a model lacking a second-order general factor showed better values for relative and absolute indices, as well as both residual metrics. Nonetheless, both models demonstrated satisfactory fit, suggesting comparable overall adequacy. Moreover, the distinctiveness of the social empowerment factor from others is strongly supported (as evidenced by the inferior fit of the three-factor solutions), which is significant given the concerns raised in earlier validation steps.

Because the hierarchical and correlated four-factor models were closely matched on the primary fit indices, a further set of comparisons was conducted to adjudicate between them. The models were first compared using the Akaike (AIC) and Bayesian (BIC) information criteria. Consistent with the primary results, both four-factor solutions produced substantially lower AIC and BIC values than the single-factor solution (AIC = 20006.09, BIC = 20107.05) and the three-factor solutions (lowest three-factor AIC = 18712.08, BIC = 18825.65), confirming the superiority of the four-factor structure. The two four-factor models nonetheless remained close, with the correlated model showing a slight advantage on both criteria (AIC = 17938.60, BIC = 18064.80) over the hierarchical model (AIC = 17966.10, BIC = 18083.89).

Given this proximity, the two nested models were compared directly using a scaled chi-square difference test [46], which is appropriate for a robust estimator and computes the difference statistic from the standard, rather than the scaled, per-model test statistics. The test indicated that the hierarchical model fit the data significantly worse than the correlated model, Δ*χ*²(2) = 34.63, *p* < .001. Thus, although the hierarchical model met conventional thresholds for acceptable fit in absolute terms, imposing the second-order structure produced a significant loss of fit relative to the correlated solution. The information criteria and the scaled difference test converge on the same conclusion: the four-factor structure is strongly supported, but the data favor a model of four correlated factors over the theoretically anticipated hierarchical one. Consequently, interpreting the four factors as correlated dimensions is empirically preferred. A general score may still be computed where a single summary index is required, but parsimony alone does not establish its psychometric adequacy, and it should be interpreted with corresponding caution. Hypothesis 1 is therefore not supported, as the four-factor conceptualization of career sustainability is clearly upheld, whereas the superiority of the higher-order specification is not confirmed in the Polish context.

Measurement invariance was tested using a multigroup approach, with the four-factor correlated model fitted under progressively more restrictive equality constraints (configural, metric, scalar, and residual). Because the *χ*² difference test is a known function of sample size and tends to flag trivial differences as significant in larger samples [47,48], invariance decisions were based primarily on changes in approximate fit indices, with ΔCFI ≤ .010, ΔRMSEA ≤ .015, and ΔSRMR ≤ .030 (loadings) or ≤.010 (intercepts and residuals) taken to indicate invariance [49,50]; the χ² difference test is nonetheless reported for transparency. Full results are presented in Table 3.

Invariance across gender (female *n* = 352, male *n* = 131; 14 cases were excluded as gender was not reported) was supported at every level. The configural model fitted the data well (CFI = .954, RMSEA = .075, SRMR = .055), and imposing equality of loadings (metric), intercepts (scalar), and residuals produced negligible changes in fit at each step (all ΔCFI ≤ .006, all ΔRMSEA ≤ .003, all ΔSRMR ≤ .001), well within the adopted thresholds; the *χ*² difference tests were correspondingly non-significant (metric Δ*χ*²(8) = 1.50, *p* = .993; scalar Δ*χ*²(8) = 10.40, *p* = .240; residual Δ*χ*²(12) = 4.47, *p* = .973). Strict residual invariance was therefore established across gender. Invariance across age was tested between Generation Z (*n* = 269) and Generation Y (*n* = 163); Generation X (*n* = 65) was excluded, as the subgroup was too small for stable multigroup estimation. The configural model again fitted well (CFI = .965, RMSEA = .064, SRMR = .055), and all subsequent constraints fell within the adopted fit-difference criteria (largest ΔCFI = −.007 at the scalar step; all ΔRMSEA ≤ .003; all ΔSRMR ≤ .006). The *χ*² difference tests were significant at the metric (Δ*χ*²(8) = 16.97, *p* = .031) and scalar (Δ*χ*²(8) = 24.60, *p* = .002) steps; however, consistent with the rationale above, these were not treated as decisive, as the accompanying changes in CFI, RMSEA, and SRMR indicated no meaningful deterioration in fit [48,49]. Residual invariance was likewise supported (Δ*χ*²(12) = 6.31, *p* = .900). Taken together, these results support Hypothesis 2: the Polish version of SCS demonstrated measurement invariance across both gender and age groups, allowing meaningful comparison, with the qualification that age invariance was established for Generations Z and Y only.

In the final step, the reliability coefficients were assessed. Cronbach’s alpha for the overall score was.81, while the subscales recorded equal or higher scores:.89,.81,.88, and.93, respectively. McDonald’s omega also achieved a satisfactory level of.93. As detailed in Table 1, removing no item increased internal stability, either for the overall score or for any of the subscales. Therefore, the SCS was deemed reliable, confirming Hypothesis 3.

## Study 2: Follow-up study

The primary objective of Study 2 was to substantiate the validity of the SCS, encompassing its criterion and discriminant validity, and its lagged associations with well-being outcomes. Additionally, by assessing the temporal stability of the SCS results, the study sought to affirm its reliability further. Thus, a series of new hypotheses were formulated in addition to testing Hypothesis 3, which was also evaluated in this study.

*H4. The SCS exhibits criterion validity in assisting career sustainability as indicated by the positive and significant correlation of career sustainability with (a) work engagement, (b) work meaning, (c) job satisfaction, and (d) work-life balance, and a negative and significant correlation with (e) job burnout.*
*H5. The SCS exhibits discriminant validity in assisting career sustainability as indicated by the higher value of the SCS’s square root of average variance extracted (AVE) than the correlation between career sustainability and (a) decent work, (b) career calling style, and (c) work-life balance.*
*H6. Career sustainability measured by SCS is prospectively associated with (a) eudaimonic well-being and (b) life satisfaction.*

The constructs to evaluate Hypothesis 4 were selected based on existing evidence and theoretical underpinnings. Notably, sustaining a career is often enhanced by perceiving one’s work as meaningful [51], engaging [52], and satisfying [53], thereby suggesting an expected positive correlation. Conversely, burnout, which diminishes perceived internal marketability and career satisfaction, tends to reduce career sustainability [3]. For Hypothesis 5, constructs that were similar yet distinct on theoretical grounds were chosen to confirm that the SCS accurately differentiates their measurement. Indeed, decent work and a sense of career calling are fundamental to career sustainability [54]; however, exploring their nuances is prudent. The rationale for Hypothesis 6 was derived from prior studies on career sustainability and evidence of the original scale’s predictive validity [29]. Data for Study 2 were collected between May 18^th^ and June 25^th^ for the first wave, and between August 12^th^ and 19^th^ for the second wave.

### Method

#### Participants and sampling.

The eligibility criteria from previous studies were applied to Study 2. A retrospective calculation determined the necessary sample size for this follow-up study’s second measurement. Using multiple regression analysis and based on results in the original study [29], the required sample size was calculated with G*Power (version 3.1.9.7). With an effect size of.19, a significance level of.05, a power of.95, and five predictors (SCS and four demographics), at least 95 individuals were needed. The target was set for a minimum of 100 valid responses to account for potential data loss. This sample pertains to the second wave of the longitudinal study. Given the planned interval of approximately seven weeks and the study’s brief nature, a 30% retention rate was anticipated, necessitating an initial sample of at least 333 participants. Considering possible data loss, the recruitment goal was set at a minimum of 360 participants.

Out of 633 individuals who started the study, 554 met the eligibility criteria, and 471 ultimately completed the survey. Study 1 and Study 2 were administered with LimeSurvey’s cookie-based duplicate-response control enabled, so that repeated participation across the two studies was prevented as far as the platform allows. After conducting attention checks, 16 participants were further excluded, yielding a final sample size of 455. Because the first wave was administered over a five-week recruitment window while the second wave was administered within a single week, the interval between measurements varied across participants, ranging from 6.86 to 11.14 weeks (M = 8.43, SD = 1.54). Sensitivity analysis, assuming α = .05 and 1 − *β* = .95, yielded critical values of.04 for regression analyses and.08 for correlation analyses.

The sample predominantly consisted of women (71.4%), with men comprising 25.5%, and the remainder not disclosing their gender. Participants had an average age of 30.7 years (*SD* = 9) and 8.9 years of work experience (*SD* = 8). The majority (67.3%) worked in office settings, while manual and combined types of work were less common, accounting for 9.5% and 23.3% of the sample, respectively. Over half of the participants were employed under an employment contract (53.8%), and a third under a civil contract (31.9%). The vast majority worked in the service sector (89.7%) and held a higher education degree (71.6%).

The data collection procedure, including informed consent and the ethical arrangements described above, mirrored that of previous studies, with several additional steps. Initially, a third attention check was introduced midway through the study, which required participants to select a specific pre-determined response to ensure their attention. Following the administration of the SCS, participants undertook a series of additional psychological tests to assess variables pertinent to Hypotheses 4 and 5.

At the study’s conclusion, participants were requested to provide their email addresses and consent to be contacted in a few weeks with an invitation to a subsequent study. Of the initial group, 352 individuals consented. These participants were subsequently invited via email to participate in a survey, which involved completing questionnaires relevant to Hypothesis 6. After the invitation, 117 participants started, and 97 completed this phase of the study. The follow-up subsample consisted predominantly of women (70.1%), with a mean age of 31.4 years (SD = 8.8) and 8.4 years of work experience (SD = 7.8). Participants who completed the follow-up (*n* = 97) did not differ significantly from those who did not (*n* = 358) in gender, χ²(1) = 0.11, p = .745, age, t(453) = 0.91, p = .363, or work experience, t(453) = −0.67, p = .506.

To link the two waves while protecting privacy, we used LimeSurvey’s token feature. The survey dataset contained each participant’s unique token but no email address; a separate file, stored on an internal university server, held the token–email pairings used to send invitations. The two waves were merged via the token, and the separate file with email addresses was deleted immediately afterward.

#### Measurement.

In this study, the Polish version of the SCS was administered, along with previously mentioned demographic questions. Additionally, the presented questionnaires were utilized.

To evaluate work engagement, the *Utrecht Work Engagement Scale* (UWES [55,56]) was utilized. This scale measures the positive cognitive and emotional experiences associated with work, including vigor, dedication, and absorption. Participants rated their responses to nine questions on a 7-point Likert scale, from *never* to *always*.

The *Work and Meaning Inventory* (WAMI) was employed to assess the sense of meaning and significance individuals find in their work (WAMI [57,58]). The WAMI evaluates work significance by exploring perceptions of work as a source of personal growth, meaningful experiences, and motivation to contribute to broader welfare. Responses were collected on a 7-point Likert scale.

Job satisfaction was measured using a shortened 5-item version of the *Brief Job Satisfaction Measure Scale II* (BJSM II [55,59]), with participants responding on a 7-point Likert scale.

Job burnout was assessed with the *Burnout Assessment Tool* (BAT [60,61]), which defines burnout as emotional and physical depletion from workplace stress. Responses were collected on a 7-point Likert scale, from *never* to a*lways*.

Decent work was evaluated using the *Decent Work Scale* (DWS [62,63]). The DWS uses 15 items to examine decent work attainment, characterized by favorable physical and interpersonal conditions, healthcare access, adequate compensation, reasonable work hours, and organizational values supporting family and social life. Responses ranged from *completely disagree* to *completely agree*.

Career calling was tested using the *calling* subscale of the *Occupational Orientation Styles* (OOS [64]), with participants rating five statements on a 7-point Likert scale.

Work-life balance was measured by a single item on a 5-point scale, asking participants to evaluate their balance directly.

Eudaimonic well-being was assessed using the *Mental Health Continuum* (MHC-SF [65]), focusing on the psychological dimension. Participants rated their experiences on a 7-point Likert scale, from *never* to *every day*.

The *Oxford Happiness Questionnaire* (OHQ-8 [66,67]) was used to evaluate participants’ life satisfaction, assessing dimensions of happiness such as life satisfaction, self-esteem, and general mood on a 7-point Likert scale.

#### Data analysis approach.

Attention checks, as described earlier, along with mandatory questions, were employed to ensure data quality. In the initial analysis phase, common method bias for variables measured in the first wave via self-report was estimated. According to the Podsakoff et al. [68] approach, a single general factor was extracted using the Maximum Likelihood method. If this factor explained less than 50% of the variance, common method bias would be deemed insignificant for interpreting the results.

Subsequently, descriptive statistics were calculated using the same metrics as in Study 1. These statistics were supplemented with Pearson correlation coefficients between all variables to examine the relationships pertinent to Hypothesis 4. The correlations needed to match the expected valence (positive or negative) and achieve statistical significance to confirm the hypothesis.

AVE was computed for each SCS dimension from the standardized factor loadings of the four-factor correlated model estimated in the Study 2 sample, as the mean of the squared loadings of that dimension’s items. Its square root was then compared with the dimension’s correlations with the variables listed in Hypothesis 5, which were expected to be lower for the hypothesis to be supported. This application departs from the Fornell–Larcker criterion [69] in its original form, which compares the square root of AVE with correlations between latent factors estimated within a single measurement model. Here the external constructs were assessed as observed scale scores, so the comparison involves correlations attenuated by measurement error in those scales. The test is therefore approximate, though conservative with respect to the correlations, and the results should be interpreted accordingly.

Lastly, data from the follow-up measurement were utilized to confirm the reliability and prospective association of the Polish version of the SCS. Initially, the correlation between the first and second measurements of the SCS was calculated. To further support Hypothesis 3, a coefficient greater than.70 was anticipated, commonly regarded as an indicator of high reliability. Subsequently, linear regression models were constructed with the SCS and control variables (demographics) as predictors, and variables related to the constructs included in Hypothesis 6 as outcomes. For Hypothesis 6 to be confirmed, significant models with the SCS as a significant predictor were expected.

### Results

Common method variance was estimated as the preliminary step in Study 2. A single general factor emerged (*χ*^2^ = 954.06; *p* < .001); however, it accounted for only 43% of the total variance among the variables, which does not meet the criteria established by Podsakoff et al. [68]. Consequently, it was concluded that common method bias did not significantly affect the results. Descriptive statistics were then evaluated and are presented in Table 4. Both general and subscale distributions deviated from normality at the scale level, as indicated by the Shapiro-Wilk test results. However, the general score and all SCS subscales exhibited mesokurtosis and displayed approximately similar distributions, as shown by the skewness metrics.

**Table 4:** Descriptive Statistics and Criterion & Discriminant Validity Analysis.

Scale	M [LL, UL 95% CI]	*α*	Kurt	Skew	*W*	1.	2.	3.	4.	5.	6.	7.	8.	9.	10.	11.
1. SCS Total	4.91 [4.83,4.99]	.81	−0.35	0.05	.99**	–										
2. SCS Happiness	4.54 [4.41,4.67]	.89	−0.33	−0.38	.98***	.81***	(.84)									
3. SCS Health	4.87 [4.74,5]	.77	−0.53	−0.4	.96***	.51***	.24***	(.49)								
4. SCS Productivity	5.73 [5.65,5.82]	.88	−0.6	0.45	.94***	.52***	.28***	.15**	(.80)							
5. SCS Social Empowerment	4.48 [4.34,4.63]	.93	−0.28	−0.69	.97***	.69***	.51***	−.06	.17**	(.91)						
6. Job engagement	4.76 [4.65,4.87]	.79	−0.56	0.24	.97***	.53***	.53***	.02	.27***	.50***	–					
7. Job satisfaction	3.58 [3.5,3.66]	.88	−0.62	−0.17	.96***	.73***	.72***	.38***	.28***	.45***	.62***	–				
8. Work meaning	3.22 [3.13,3.31]	.94	−0.24	−0.9	.97***	.70***	.67***	.04	.17**	.80*	.61***	.66***	–			
9. Burnout	2.32 [2.27,2.37]	.82	0.26	0.51	.99***	−.68***	−.52***	−.59***	−.34***	−.29**	−.43***	−.70***	−.42***	–		
10. Decent work	3.21 [3.15,3.27]	.83	−0.2	−0.11	.99*	.47***	.43***	.51***	.15**	.12**	.18**	.49***	.23***	−.51***	–	
11. Career calling	3 [2.92,3.08]	.82	−0.26	−0.34	.98***	.7***	.71***	.25***	.23***	.53***	.55***	.76***	.72***	−.55***	.46***	–
12. WLB	3.44 [3.36,3.53]	–	−0.4	−0.24	.89***	.4***	.34***	.52***	.13**	.05	.10*	.38***	.16**	−.52***	.54***	.33***

*Note*. *N* = 455. *M* = mean; *LL* = lower limit; *UL* = upper limit; 95% CI = 95% confidence interval; *Kurt* = kurtosis; *Skew* = skewness; *W* = Shapiro-Wilk test results; *α* = Cronbach’s alpha; Diagonals present square root of AVE values for factors.

*p < .05. **p < .01. ***p < .001.

Pearson correlation coefficients were used to evaluate Hypothesis 4. All variables of interest demonstrated significant correlations with expected valence and moderate to moderately strong magnitudes, significantly exceeding the minimal detectable effects indicated by the sensitivity analysis. Greater work engagement, job satisfaction, and work meaning were associated with higher career sustainability, while greater job burnout was linked to lower career sustainability, consistent with theoretical predictions. Therefore, the data supported Hypothesis 4, affirming the criterion validity of the Polish SCS.

The discriminant validity of the Polish version of the SCS was evaluated as outlined in Hypothesis 5. The square root of AVE for all four subscales ranged from.49 (Health) to.91 (Social Empowerment). For all scales except Health, correlations with Decent Work, Career Calling, and WLB were lower than these values, indicating satisfactory discriminant validity. For the Health subscale, however, the square root of AVE was low in absolute terms, and its correlations with Decent Work and WLB were of comparable magnitude. Discriminant validity therefore cannot be claimed for this dimension on the present evidence. Hypothesis 5 thus received partial support: it was supported for Happiness, Productivity, and Social Empowerment, but not for Health.

Finally, the prospective association was assessed as outlined in Hypothesis 6. Two regression models yielded significant results, as detailed in Table 5. The performance of the significant models, as indicated by the *R*^2^ values, was.25 for predicting Well-being and.40 for Life Satisfaction, far exceeding the minimal detectable effect as estimated through sensitivity analysis. The overall SCS score itself significantly predicted Eudaimonic Well-being (*b* = .69 [.41;.96]) and Life Satisfaction (*b* = .79 [.58; 1]). During the analysis, the assumptions for linear regression were met, as evidenced by the normality of outcome variables (W = .98, ns; W = .97, ns; for Well-being and Life Satisfaction respectively) and the absence of linearity assumption violations (*F* = 1.49, *ns*; *F* = .78, *ns*) or autocorrelation among observations (*DW* = 2.39, *ns*; *DW* = 1.98, *ns*).

**Table 5:** Regression Models for Prospective Association Analysis.

Predictor:	Original model: *b* [LL, UL 95% CI]		Robustness check (Life Satisfaction)		
	Well-being	Life Satisfaction	*SE* (HC3)	*p* (HC3)	Bootstrap 95% CI
(Intercept)	0.39 [-2.22, 3]	1.08 [-0.95, 3.12]	1.24	.384	[−1.03, 3.63]
Career Sustainability	0.69 [0.41, 0.96]	0.79 [0.58, 1]	0.13	<.001	[0.55, 1.02]
Control: gender	0.01 [-0.42, 0.44]	0.19 [-0.15, 0.53]	0.18	.295	[−0.15, 0.55]
Control: age	-0.01 [-0.09, 0.06]	-0.03 [-0.09, 0.03]	0.03	.365	[−0.10, 0.02]
Control: job experience	0 [-0.09, 0.08]	0.02 [-0.04, 0.09]	0.04	.505	[−0.04, 0.10]
Control: highest education	-0.33 [-0.89, 0.23]	0.06 [-0.37, 0.5]	0.20	.756	[−0.33, 0.45]
Model evaluation:					
*F*	6.07***	11.75***			
*R*²	0.25	0.40			
*W* (outcome)	0.98	0.97			
*W* (residuals)	0.98	0.96*			
*GQ* Goldfeld-Quandt	1.42	2.11**			
*F*_RAIN_	1.49	0.78			
*DW*	2.39	1.98			

*Note*. *N* = 97. *b* = regression coefficient; *LL* = lower limit; *UL* = upper limit; 95% CI = 95% confidence interval; *SE* (HC3) = heteroskedasticity-consistent standard error; *p* (HC3) = heteroskedasticity-consistent p-value; Bootstrap 95% CI = percentile confidence interval from 2,000 case resamples; *F* = F-statistic for model significance; *R*² = coefficient of determination; *W* (outcome) = Shapiro-Wilk test for normality of the outcome variable; *W* (residuals) = Shapiro-Wilk test for normality of residuals. *GQ* = Goldfeld-Quandt test for homoscedasticity; *F*_RAIN_ = Rainbow test for overall model linearity; *DW* = Durbin-Watson test for autocorrelation in residuals.

*p < .05. **p < .01. ***p < .001.

However, several issues were identified with the Life Satisfaction model, notably signs of heteroskedasticity (*GQ* = 2.11, *p* < .01) and deviations from normality in the residuals (*W* = .96, *p* < .05). To investigate the underlying causes, the data were analyzed for potential outliers. Neither Studentized residuals (>3) nor Cook’s distances (>1) indicated the presence of outliers. To formally address these violations, the model was re-estimated using HC3 heteroskedasticity-consistent standard errors, and coefficient confidence intervals were additionally bootstrapped (2,000 resamples) to avoid reliance on the normality assumption, as reported in Table 5. The career sustainability coefficient remained significant and of comparable magnitude under both checks (b = .79, HC3 p < .001, bootstrap 95% CI [.55, 1.02]), closely matching the original estimate and confidence interval. As the robustness checks confirmed the original result was not an artifact of the identified violations, these data support Hypothesis 6.

Lastly, test-retest reliability was assessed to estimate the temporal stability of the Polish SCS. The scores from two time points for each participant were correlated, yielding a significant (*p* < .001) and high correlation coefficient (*r* = .81). As the Pearson coefficient reflects rank-order stability and is insensitive to systematic mean-level change between measurements, absolute agreement was additionally assessed using a two-way random-effects intraclass correlation coefficient for single measurements [70], ICC(2,1) =.80, 95% CI [.72,.86]. The close correspondence between the two coefficients indicates that the stability of SCS scores was not accompanied by systematic mean-level change. Furthermore, the Cronbach’s alpha values ranged from satisfactory to exceptional (.77 to.93), reinforcing the reliability of the Polish SCS. This evidence further supports Hypothesis 3.

## Discussion

This study focused on developing and validating the Polish version of the SCS, a psychological test designed to measure subjective career sustainability following the theory proposed by Russo et al. [8]. To achieve this, a pilot study followed by two main studies were conducted, utilizing a mixed-methods approach with observational and longitudinal designs. The development and validation process confirmed the feasibility of transferring the concept of sustainability to a new cultural context while maintaining its theoretical four-factor structure. The SCS proved a reliable and valid instrument, encompassing criterion and discriminant validity and lagged associations with well-being outcomes. The data supported all hypotheses but Hypothesis 1, although Hypothesis 5 received only partial support. Consequently, this study enriches the field of career psychology by introducing a robust measurement method and demonstrating the applicability of career sustainability within a previously untested cultural context. Most importantly, this study provides further evidence supporting the novel career sustainability model, which incorporates a social empowerment dimension. It also lends credibility to the assertion that Russo’s sustainability model is transferable across the cultural contexts.

The career sustainability model advanced by Russo et al. [8,29] holds that happiness, health, productivity, and social empowerment are not merely co-occurring outcomes but converge on a single overarching construct, echoing De Vos et al.‘s [7] conception of sustainable careers as an integrated person-level state rather than a loose bundle of indicators. This theoretical premise motivated Hypothesis 1 and its expectation of a hierarchical structure in which one general factor underlies the four dimensions. The data only partly bear this out. The clearest and most robust finding was that the four dimensions are empirically distinct yet related, thereby affirming the multidimensional core of the model. At the same time, their organization under a common higher-order factor also received empirical support, although the hierarchical specification showed a less optimal fit than the model with four correlated factors. This pattern suggests that career sustainability can be interpreted in two complementary ways. On the one hand, the higher-order model supports the possibility of considering career sustainability as a broader overarching construct. However, in the present Polish context, the four correlated-factor solution appears to provide a more accurate representation of the data, suggesting that health, happiness, productivity, and social empowerment may operate as related but partially autonomous facets. Such a reading aligns with conceptualizations that treat career sustainability as an emergent property arising from the interplay of distinct components—health, happiness, productivity, and social empowerment—each shaped by different personal and contextual forces and capable of developing, or eroding, on its own [7,22]. Accordingly, the four subscale scores are to be preferred whenever the aim is to examine how specific aspects of career sustainability relate to their antecedents, outcomes, or contextual conditions, since aggregation may mask the divergent ways in which the facets operate. The total score should be treated as a broad summary measure and interpreted with caution, for example in brief screening, descriptive purposes, or as a control variable. This qualification concerns the empirical basis for aggregation in the present samples rather than the theoretical status of the higher-order construct, which the current data do not adjudicate conclusively.

Although concerns about the social empowerment factor were raised by participants during the FGIs, the quantitative data demonstrated that this factor is distinct from others, independent of related constructs, and reliably measured. A plausible explanation for the concerns is that participants perceiving low social empowerment found discussing this aspect of their careers challenging. Aligning career development with SDGs on decent work and dignity is essential, particularly for employees in emerging markets, poorer economies, and the gig workforce, to promote universal, sustainable, and equitable development [71]. Integrating sustainability training with the SDGs has been shown to impact sustainable career development positively [72] and also yields positive effects on organizations, improving employee satisfaction and productivity, reducing environmental impact, and enhancing the organization’s reputation [73].

Measurement invariance held across gender, and full invariance was also supported between Generations Z and Y, indicating that the SCS functions equivalently for these groups. Invariance could not be tested for Generation X, as the subgroup was too small for stable estimation; this limitation may be relevant given that generational cohorts can differ in the cultural values they emphasize, such as hedonism, traditionalism, and universalism [74]. Generation X has been characterized as prioritizing traditional career paths and placing less emphasis on work-life balance [75,76]. Whether such generational differences affect the measurement properties of the scale across older cohorts remains an open question, particularly as younger generations tend to favor non-linear careers and show greater sensitivity to social and environmental impact [77,78].

Our findings from both studies corroborate the reliability of the SCS and suggest that individuals’ perceptions of career sustainability exhibit a high degree of stability over an interval averaging approximately eight weeks. Given the potential role of career shocks in career development [6], future studies could utilize our scale to investigate how such events impact career sustainability. The results reinforce the scale’s validity as well, supporting theories that perceiving work as meaningful, engaging, and satisfying enhances career sustainability. Consistent with Barthauer et al. [3], burnout was found to reduce internal marketability and career satisfaction, both vital to career sustainability. While decent work, career calling, and WLB are essential elements [54], our results confirm their distinctiveness from career sustainability.

### Theoretical contribution

This research advances the field of career psychology by providing evidence for the Polish cultural adaptation and validation of the SCS, demonstrating that the core dimensions of career sustainability—happiness, health, productivity, and social empowerment—are recognizable and measurable within a new cultural context. A major contribution of this paper is the comparison of Russo et al.‘s [29] four-factor model with a three-factor model, where social empowerment is subsumed. Our results clearly favor the four-factor structure over the three-factor alternative, with both the correlated and the hierarchical four-factor specifications outperforming every three-factor solution, showing that happiness, health, productivity, and social empowerment are linked but distinct in defining career sustainability. The successful adaptation and validation of the scale in the Polish context highlight the need for universally applicable career models that accommodate cultural differences, aiding in global comparative studies and the formulation of international career development strategies [13]. This deepens theoretical insights and enhances the efficacy and relevance of career interventions across diverse cultural landscapes [26].

This study offers empirical support for the theoretical construct of career sustainability as a non-linear sequence of individual work experiences facilitating the development of health (mental and physical well-being), happiness (career satisfaction and success), productivity (perceived performance and employability), and social empowerment (feeling part of society and ethically addressing societal needs) [8]. By validating this construct in a new cultural context, this study underscores the need to shift away from an individualistic, neoliberal perspective on career sustainability and towards one grounded in collective dignity, striving to respect and promote the intrinsic value of all individuals and the planet itself [79]. Sustainable career development requires an approach that moves towards the construction of inclusive and sustainable contexts and futures, promoting social justice and preserving the world’s human and environmental resources [23,71,80].

### Limitations and future studies guidelines

While this study provides valuable insights into the construct of career sustainability in a Polish context, it is important to acknowledge certain limitations. Across all three samples, women, service-sector and white-collar employees, and individuals with higher education were each represented at over 70%, limiting the generalizability of the findings. This homogeneity is particularly relevant for the productivity and social empowerment dimensions, which may carry different meanings for blue-collar or lower-autonomy workers, potentially affecting both the relevance of specific items and the observed factor structure in more occupationally diverse populations. Future studies should aim to include a more diverse sample, including blue-collar workers and individuals from different socioeconomic backgrounds. Given the dynamic and evolving nature of career sustainability [7], future research should adopt longitudinal designs to track changes in perceptions of career sustainability over time, extending beyond the interval examined here. The absence of a Time-1 baseline for the outcomes means the findings represent lagged associations rather than the prediction of change, and future longitudinal work should include baseline outcome measures. A further limitation concerns the average variance extracted for the Health dimension, which was comparatively low in both studies and particularly so in Study 2. This indicates limited convergence among the Health items and, in Study 2, precluded a claim of discriminant validity for this dimension relative to Decent Work and work–life balance. Replication with a longer Health subscale, and with external constructs modelled as latent variables within a common measurement model, would allow a more definitive assessment. Furthermore, future investigations could explore the individual perceptions of career sustainability in relation to the broader context of the Sustainable Career Ecosystem [81], by examining the interplay between individuals’ perceptions with the broader societal, higher education institutions, and organizational initiatives aimed at promoting sustainable development.

## Conclusions

This study provides preliminary evidence for the Polish adaptation of the SCS, supporting its psychometric properties in Polish worker samples. The findings demonstrate that the Polish version of the SCS retains the theoretical structure proposed by Russo et al. [8], consisting of four interrelated yet distinct dimensions: happiness, health, productivity, and social empowerment. In this sample, these dimensions reflect key components of sustainable careers, suggesting the model’s relevance may extend beyond the context in which it was developed. The study offers initial support for the novel sustainable career model in a new cultural context, contributing to theoretical understanding and to the cross-cultural study of sustainable careers. Further research with independent, representative, and occupationally diverse samples is needed before broader conclusions about the cross-cultural applicability of the scale can be drawn.

## Pre-registration

The study was pre-registered on the Open Science Framework (https://osf.io/cqbh9). No substantial discrepancies were identified between the registered protocol and the actual study conducted, aside from a wording revision to Hypothesis 6, in response to peer review. The core analyses were conducted as planned: the pilot EFA and reliability analyses, the focus group interviews; in the primary studies, the descriptive statistics, power/sensitivity analyses, reliability estimates, the confirmatory factor analysis comparing the competing models, measurement invariance, the AVE-based discriminant validity assessment, common method bias estimation, the criterion-validity correlations, the predictive-validity regressions (here as lagged associations), and test–retest reliability. Three analyses were not part of the registered protocol: the comparison of models using AIC, BIC, a scaled chi-square difference test, the parallel analysis, and expanded factor-structure reporting in S1 File, all added during revision.

## Supporting information

S1 File Additional data analyses. (DOCX)
