# Supplementary material for: Polish adaptation and validation of the sustainable career scale
Source: PLoS One. 2026 Sep 8;21(9):e0357496. doi: 10.1371/journal.pone.0357496 (PMC13552746; doi:10.1371/journal.pone.0357496)
Supplement: Supplementary file 1 [file pone.0357496.s001.docx]

**S1 File**

**Additional data**	**analyses**

**Focus Group Interviews results**

Table S1 presents item-level comprehension data alongside representative participant statements for each item, organized by coding category. For each item, the table reports the number of participants who rated it as clear, expressed uncertainty about its clarity, and reported knowing how to formulate a response. Qualitative statements are presented separately for two coding categories: item comprehension, which captures difficulties related to the wording, scope, or referent of a specific item; and construct understanding, which captures broader reflections on the expected content of the questionnaire or participants' lay definitions of sustainable career.

Comprehension ratings were generally high for the Happiness (items 1–3) and Productivity (items 7–9) subscales, with the majority of participants endorsing clarity across these items. The Health subscale (items 4–6) generated item comprehension concerns related to ambiguity of valence (item 4) and scope (item 6). The Social Impact subscale (items 10–12) received the lowest comprehension ratings overall, with item 12 endorsed as clear by only four out of eight participants. Qualitative statements for this subscale reflected both item-level difficulties — particularly the perceived overlap between items — and construct-level concerns regarding the relevance of the social impact dimension to participants' occupational contexts.
FGI findings resulted in revisions to the questionnaire. The questionnaire instructions were expanded to define career explicitly as a sequence of work experiences, unfolding across the life span, directly addressing the difficulties reported by participants with Happiness and Social Empowerment subscales. In items 10 to 12, the term *work* was replaced with *career* for consistency across the questionnaire; item 11 was reworded from “quality of life” to a more accessible formulation (“helping people live better”), while item 12 replaced “well-being” with “happiness” to reduce conceptual overlap between items. In item 2, the verb tense was adjusted from past ("has evolved") to present continuous ("is developing") to reflect the ongoing nature of career, addressing participants' concerns that the original phrasing implied a completed trajectory. Item 6 was revised to include an explicit frequency qualifier ("often") and to clarify that the inability to concentrate extends beyond the workplace, resolving ambiguity about the scope of work-related fatigue. Item 9 was reworded from "I believe I am good at my job" to "In my opinion, I am good at my job" to anchor the item in a personal evaluative judgment rather than general conviction, following participants' reflections on whether the belief was internally or externally grounded.

**Table S1**

*Item-Level Comprehension Ratings and Participant Responses by Item and Category (N = 8)*

| **No.** | **Subscale** | **Yes** | **Unsure** | **Knew** | **Item comprehension** | **Construct understanding** |
| --- | --- | --- | --- | --- | --- | --- |
| 1 | Happiness | 7 | 1 | 5 | “I didn’t know in what sense ‘satisfied with life,’ or in what sense [my career] allows me to be—is it through money, job satisfaction, or work–life balance?” | “I understand this as separating work from private life.” |
| 2 | Happiness | 8 | 0 | 5 | “I’m not sure how to answer, because I compare myself both to people with a better career and to those in a worse situation.” | “The question gives a sense that the career can no longer change, which triggers negative feelings.” |
| 3 | Happiness | 8 | 0 | 7 | “I felt a lack of context—a workaholic could answer the same way as me, looking at completely different factors.” | — |
| 4 | Health | 7 | 1 | 6 | “I didn’t know whether it had a negative character (e.g., from stress) or a positive one (e.g., I come up with an idea).” | — |
| 5 | Health | 8 | 0 | 5 | “Mental health, physical health, or both?” | — |
| 6 | Health | 7 | 1 | 5 | “I was unsure what I can’t concentrate on—tasks at work or in general?” | — |
| 7 | Productivity | 5 | 1 | 5 | “On one hand, yes—I know the procedures; on the other, managers often question my opinion, which makes me doubt my own competence.” | — |
| 8 | Productivity | 8 | 0 | 6 | “I would have liked to specify who and what was responsible for my preparation.” | — |
| 9 | Productivity | 8 | 0 | 7 | “I found myself reflecting on the source of this belief—internal conviction, or based on others’ opinions?” | — |
| 10 | Social Empowerment | 5 | 3 | 4 | “It seems this is reserved only for professions such as educator, therapist, psychologist, or doctor.” | “In what sense ‘better’? Morally? In terms of competence? Or hedonistically—like giving them good coffee?” |
| 11 | Social Empowerment | 5 | 3 | 3 | “I’m not sure what counts as improving quality of life, and I don’t know whether my work offers that.” | “Improving life by selling better cars or smartphones—yes. But not by running a café.” |
| 12 | Social Empowerment | 4 | 4 | 3 | “I don’t know how this question differs from the previous one.” | “I feel my work has little to do with the well-being of the community.” |

*Note.* “Clear” = item was rated as clear; “Unsure” = participant was unsure about item clarity; “Knew” = number of participants who reported knowing how to respond (out of 8). Participant quotes were translated from Polish by the authors. Dashes indicate no statements recorded for that category.

**EFA results from the Pilot Study**

Next, an EFA was conducted. Initial tests verified the data's suitability for factor analysis, with Bartlett's Test of Sphericity (χ² = 1053.87; df = 66; p < .001) and the Kaiser-Meyer-Olkin measure (.82) both supporting this. A four-factor solution was extracted using the minimum residuals method with oblimin rotation, consistent with the theoretical structure of the SCS. The full pattern matrix, including all factor loadings and cross-loadings, is reported in Table S1. The solution displayed a clear simple structure: each item loaded substantially on its intended factor, with target loadings ranging from .66 to .97, while all cross-loadings were negligible (≤ .20). The majority of target loadings met the criterion for an excellent value (> .71; Comrey & Lee, 1992), and the remainder were very good (> .63). The communalities (h²), also reported in Table S2, ranged from .47 to .94, indicating that the four-factor solution accounted for a substantial proportion of each item's variance. The lowest value, for a Health item, remained acceptable, and no communality approached a level indicative of an estimation problem. On this basis, we proceeded with further validation of the Polish version of the SCS (available at the Open Science Framework repository, https://osf.io/2n4r5/files/32va5), which initially indicated satisfactory psychometric properties.

**Table S2**

Exploratory Factor Analysis Pattern Matrix for the Polish Sustainable Career Scale (Pilot Study)

|  | Factor loadings | | | |  |  |
| --- | --- | --- | --- | --- | --- | --- |
| **Item** | Happiness | Health | Productivity | Social Empowerment |  |  |
| 1 | 0.96 | -0.01 | 0 | -0.04 |  |  |
| 2 | 0.86 | 0.05 | -0.02 | 0.06 |  |  |
| 3 | 0.68 | 0.01 | 0.1 | 0.2 |  |  |
| 4 | 0.05 | 0.66 | -0.01 | -0.02 |  |  |
| 5 | -0.1 | 0.88 | -0.02 | 0.09 |  |  |
| 6 | 0.12 | 0.84 | 0.02 | -0.08 |  |  |
| 7 | -0.05 | -0.02 | 0.96 | 0 |  |  |
| 8 | -0.02 | 0 | 0.91 | 0.02 |  |  |
| 9 | 0.08 | 0.02 | 0.86 | -0.03 |  |  |
| 10 | 0.14 | 0.03 | 0.06 | 0.78 |  |  |
| 11 | -0.03 | 0.06 | 0 | 0.97 |  |  |
| 12 | 0.01 | -0.08 | -0.02 | 0.94 |  |  |

*Note*. N = 100. Factor loadings were obtained using the minimum residuals extraction method with oblimin (oblique) rotation. Health items were reverse-scored prior to analysis.

The number of factors to retain was further examined through the eigenvalues of the correlation matrix and a parallel analysis (Horn, 1965). The eigenvalues of the first four factors were 5.21, 2.51, 1.39, and 0.52, with all subsequent values approaching or falling below zero (see Table S2). Applying the Kaiser criterion (eigenvalue > 1) would suggest a three-factor solution, as the fourth eigenvalue fell below unity. However, the Kaiser criterion is widely recognised to under-extract factors and to rest on an arbitrary threshold (Hayton et al., 2004), and was therefore not used as the decisive rule, given the exploratory character of the pilot analysis, the small sample size, and the strong theoretical justification for four dimensions. Parallel analysis, which retains factors whose observed eigenvalues exceed those generated from random data of equivalent dimensions and is regarded as a more accurate criterion, supported the retention of four factors: as shown in Figure S1, the observed eigenvalues exceeded their simulated counterparts for the first four factors and fell below them thereafter. The fourth factor, although modest in magnitude, was supported by the parallel analysis and is consistent with the theoretical four-dimensional model of the SCS (Russo et al., 2023, 2025), providing a sufficient empirical basis to proceed to confirmatory testing.

**Figure S1**

Parallel Analysis Scree Plot for the Polish Sustainable Career Scale (Pilot Study)

*Note*. N = 100. The solid line represents the eigenvalues obtained from the actual data; the dashed and dotted lines represent eigenvalues derived from simulated and resampled random data, respectively. Factors are retained where the observed eigenvalues exceed those of the random data, supporting a four-factor solution. Analysis conducted using the minimum residuals extraction method.

The oblimin rotation permitted the factors to correlate, and the resulting inter-factor correlations are reported in Table S3. The correlations were generally low to moderate, ranging from −.13 to .67, indicating that the four dimensions are related yet empirically distinct. The strongest association was between Happiness and Social Empowerment (.67), while Happiness also correlated moderately with Health (.40) and Productivity (.36). Health showed the weakest associations with the remaining dimensions, including a small negative correlation with Productivity (−.13). This pattern of moderate intercorrelations is consistent with the conceptualisation of career sustainability as a set of related but separable components rather than a single undifferentiated construct, and supports the use of an oblique rotation.

**Table S3**

Inter-Factor Correlations from the Exploratory Factor Analysis of the Polish Sustainable Career Scale (Pilot Study)

| Factor | Social empowerment | Productivity | Happiness |
| --- | --- | --- | --- |
| Social empowerment | - |  |  |
| Productivity | 0.3 | - |  |
| Happiness | 0.67 | 0.36 | - |
| Health | 0.15 | -0.13 | 0.4 |

*Note*. N = 100. Correlations were obtained from a four-factor solution using the minimum residuals extraction method with oblimin (oblique) rotation. Health items were reverse-scored prior to analysis.

It should be emphasised, however, that the presented results are exploratory and warrant cautious interpretation given the small pilot sample. They served to determine whether the adapted scale was suitable for the more rigorous confirmatory testing that followed, rather than to establish its factor structure definitively.

**References**

Hayton, J. C., Allen, D. G., & Scarpello, V. (2004). Factor retention decisions in exploratory factor analysis: A tutorial on parallel analysis. *Organizational Research Methods*, *7*(2), 191–205. https://doi.org/10.1177/1094428104263675

Horn, J. L. (1965). A rationale and test for the number of factors in factor analysis. *Psychometrika*, *30*(2), 179–185. https://doi.org/10.1007/BF02289447

Russo, A., Valls-Figuera, R. G., Zammitti, A., & Magnano, P. (2023). Redefining 'Careers' and 'Sustainable Careers': A qualitative study with university students. *Sustainability*, *15*(24), 16723. https://doi.org/10.3390/su152416723

Russo, A., Zammitti, A., Zarbo, R., Magnano, P., & Santisi, G. (2025). The Sustainable Career Scale (SCS): Development, validity, reliability and invariance. *Journal of Global Responsibility*. https://doi.org/10.1108/JGR-07-2024-0127
