# Supplementary material for: Polish adaptation and validation of the sustainable career scale
Source: PLoS One. 2026 Sep 8;21(9):e0357496. doi: 10.1371/journal.pone.0357496 (PMC13552746; doi:10.1371/journal.pone.0357496)
Supplement: Supplementary file 3 [file pone.0357496.s003.docx]

We thank the Reviewer for the careful reading of our manuscript and for the constructive comments provided throughout the review process. We have addressed each of the remaining points below, indicating where changes have been made in the manuscript. We are grateful for the attention this review has given to the accuracy and clarity of our reporting, and we hope that the revised manuscript now meets the criteria for publication.

**Comment 1.1.** 1. Ethics approval chronology. The most important remaining issue concerns the ethics approval date. The manuscript reports that the data were collected between March and August 2024, whereas the stated ethical approval was issued on 17 March 2026.

Please clarify whether “2026” is a typographical error. If so, please provide the correct date and revise it consistently in the manuscript and submission information. If the approval was genuinely issued in 2026, please explain what prospective ethical approval, exemption, or institutional authorization covered the participant recruitment and data collection conducted in 2024.

**Response**: We thank the Reviewer for this question, which gives us the opportunity to set out the chronology of ethical oversight for this project in full. We recognize that our previous response described the ethical status of the study without making the sequence of events explicit, and we are grateful for the chance to correct that omission here.

The date is not a typographical error. Data collection took place between March and August 2024, and the Committee's decision (Opinion no. 07/03/2026) was issued on 17 March 2026. The sequence was as follows.

At the time the research was conducted, prospective review by an ethics committee was not required for a study of this type. Under the Regulations of the Ethics Committee for Research Projects of Adam Mickiewicz University in Poznań, not all research requires committee review; minimal-risk, anonymous, non-clinical survey research conducted with consenting adults, involving no sensitive or potentially harmful content and no experimental manipulation, falls outside the categories for which prospective review is mandated. This position is consistent with the broader regulatory context in Poland, where non-medical psychological research does not fall under the statutory framework governing medical experiments, and with international norms treating anonymous surveys of competent adults on non-sensitive topics as minimal-risk research eligible for exemption or expedited review.

The absence of a requirement for committee review did not mean the research was conducted without ethical governance, and the relevant safeguards were already described in the submitted manuscript. The Method section of the pilot study states that informed consent was obtained via an online form from all participants before the pilot and each subsequent study, that participants were fully informed about the nature and purpose of the research and consented to the use of their data for publication, and that all procedures adhered to the tenets of the Declaration of Helsinki; the procedure section adds that participants were presented with an introductory page outlining the study details and their rights, and the Study 1 and Study 2 Method sections confirm the same consent procedure. As described in Study 2 and clarified in response to the Reviewer's earlier comment, the two waves were linked by a system-generated token rather than by email address, with token–email pairings held separately and deleted immediately after merging, leaving no identifying information in the archived dataset.

Following completion of the project, and in the course of preparing the manuscript for submission to PLOS ONE, we sought to document the study's ethical standing formally in order to meet the journal's requirements. The Regulations of the Ethics Committee for Research Projects of Adam Mickiewicz University in Poznań provide for retrospective review of research conducted within the preceding four years. We therefore submitted the full research programme — comprising the focus group interviews, the pilot study, Study 1, and Study 2 — to the Committee for evaluation. Our application stated explicitly that data collection had already been concluded. The Committee reviewed the submitted materials with that fact before it and issued a positive opinion regarding the ethical aspects of the research described in the application. The retrospective submission was voluntary; we sought it because we regard documented ethical oversight as valuable in itself and appropriate to current expectations of PLOS. Moreover, the Committee's assessment was made in full knowledge that the study had been completed, so the opinion constitutes a substantive evaluation of the research as actually conducted rather than a pro forma endorsement.

We have revised the manuscript so that this sequence is stated explicitly rather than inferable from the decision number alone. The Method section now reads:

Prospective ethical review was not required for this study, which involved minimal-risk, anonymous survey research with consenting adults and no sensitive or potentially harmful content. The project was subsequently submitted for retrospective review and received a positive opinion from the Ethics Committee for Research Projects of Adam Mickiewicz University in Poznań (Opinion no. 07/03/2026, issued 17 March 2026); the application covered the full research programme and stated that data collection had already been concluded.

We have also added cross-references in Study 1 and Study 2 confirming that the ethical arrangements described in the pilot study applied to those studies as well, and we have updated the submission information so that it is fully consistent with the manuscript.

The Committee's opinion has already been provided in the submission system. We addressed the ethical status of the study in our previous response, and we apologize if our earlier explanation was not sufficiently clear as to the sequence of events; we hope the account above resolves the matter fully.

**Comment 1.2.** 2. Interpretation of the total SCS score: The revised analyses indicate that the four correlated-factor model fits better than the hierarchical model. Therefore, the interpretation of the total SCS score should be further qualified.

Please avoid implying that parsimony alone provides sufficient psychometric justification for the total score. The manuscript should state more clearly that the four subscale scores may be preferable when examining dimension-specific relationships, whereas the total score should be interpreted cautiously as a broad summary measure.

**Response**: We thank the Reviewer for this observation, which we agree required a clearer statement in the manuscript. We have revised the interpretation of the total SCS score in both places where it was addressed.

In the Results of Study 1, the previous wording stated that a general score "remains defensible on parsimony grounds," which, as the Reviewer notes, could be read as treating parsimony as sufficient psychometric justification for aggregation. That was not our intention, and the sentence has been revised to make the limitation explicit. The passage now reads:

Consequently, interpreting the four factors as correlated dimensions is empirically preferred. A general score may still be computed where a single summary index is required, but parsimony alone does not establish its psychometric adequacy, and it should be interpreted with corresponding caution.

In the Discussion, we have reversed the order of the guidance so that the subscale scores, rather than the total score, are presented as the preferred option, and we have restored the substantive reason for that preference. The revised passage reads:

Accordingly, the four subscale scores are to be preferred whenever the aim is to examine how specific aspects of career sustainability relate to their antecedents, outcomes, or contextual conditions, since aggregation may mask the divergent ways in which the facets operate. The total score should be treated as a broad summary measure and interpreted with caution, for example in brief screening, descriptive purposes, or as a control variable. This qualification concerns the empirical basis for aggregation in the present samples rather than the theoretical status of the higher-order construct, which the current data do not adjudicate conclusively.

The final sentence distinguishes the practical scoring recommendation, which follows directly from the model comparison, from the broader theoretical question of whether career sustainability constitutes a higher-order construct. The present findings speak to the former; a single comparison in one national sample does not resolve the latter.

In implementing these changes, we reviewed the manuscript for any remaining statements that could be read as inconsistent with the revised interpretation. This identified a sentence in the Theoretical Contribution section which, although concerned with the four-factor versus three-factor comparison rather than the correlated versus hierarchical comparison, could nonetheless have been misread as asserting the superiority of the hierarchical solution. It now reads:

Our results clearly favor the four-factor structure over the three-factor alternative, with both the correlated and the hierarchical four-factor specifications outperforming every three-factor solution, showing that happiness, health, productivity, and social empowerment are linked but distinct in defining career sustainability.

**Comment 1.3.** 3. Convergent and discriminant validity: Please moderate the claims concerning the convergent and discriminant validity of the Health dimension. The relatively low square root of AVE, particularly in Study 2, should be acknowledged as a limitation.

The manuscript should also clarify how AVE was calculated in Study 2 and explain the limitations of applying the Fornell–Larcker criterion when the external constructs were not all assessed within a common latent-variable measurement model.

**Response:** We thank the Reviewer for these observations, which we have addressed in three respects: the calculation of AVE, the limitations of the Fornell–Larcker comparison as applied here, and the strength of the claims made for the Health dimension.

Regarding the calculation, the manuscript did not previously state how AVE was obtained in Study 2. The analytical strategy section now specifies this and explains the limitations of applying the criterion when the external constructs were not assessed within a common latent-variable measurement model:

AVE was computed for each SCS dimension from the standardized factor loadings of the four-factor correlated model estimated in the Study 2 sample, as the mean of the squared loadings of that dimension's items. Its square root was then compared with the dimension's correlations with the variables listed in Hypothesis 5, which were expected to be lower for the hypothesis to be supported. This application departs from the Fornell–Larcker criterion (Fornell & Larcker, 1981) in its original form, which compares the square root of AVE with correlations between latent factors estimated within a single measurement model. Here the external constructs were assessed as observed scale scores, so the comparison involves correlations attenuated by measurement error in those scales. The test is therefore approximate, though conservative with respect to the correlations, and the results should be interpreted accordingly.

Regarding the Health dimension, we agree that our previous wording was too permissive. The Results of Study 2 previously described the correlations of Health with Decent Work and WLB as suggesting "a partial issue" with the independence of the subscale, which understated the position. The passage now reads:

For the Health subscale, however, the square root of AVE was low in absolute terms, and its correlations with Decent Work and WLB were of comparable magnitude. Discriminant validity therefore cannot be claimed for this dimension on the present evidence. Hypothesis 5 thus received partial support: it was supported for Happiness, Productivity, and Social Empowerment, but not for Health.

We note that the same issue, although less pronounced, is present in Study 1, where the square root of AVE for Health was also the lowest of the four dimensions. Since the AVE values constitute the evidence bearing on the convergence of items within each dimension, this qualifies the convergent as well as the discriminant validity of the Health subscale, and we have added a corresponding statement to the Results of Study 1:

While each value exceeded the corresponding inter-factor correlations, the value for Health was comparatively low, indicating limited convergence among its items.

We have also acknowledged the low average variance extracted as a limitation, as the Reviewer requests:

A further limitation concerns the average variance extracted for the Health dimension, which was comparatively low in both studies and particularly so in Study 2. This indicates limited convergence among the Health items and, in Study 2, precluded a claim of discriminant validity for this dimension relative to Decent Work and work–life balance. Replication with a longer Health subscale, and with external constructs modeled as latent variables within a common measurement model, would allow a more definitive assessment.

Finally, we have added Fornell and Larcker (1981) to the reference list, and have standardized the use of validity terminology across the manuscript.

**Comment 1.4.** 4. Study 2 reporting: Please correct or clarify the following points:

- The power analysis refers to four predictors, whereas the regression table appears to include the SCS and four demographic control variables.

- Statistical power should be reported as 1 − β = .95 rather than β = .95.

- Please report the actual follow-up interval, because the stated data-collection dates suggest that it may not have been exactly seven weeks for every participant.

- Please provide brief statistical details supporting the statement that the follow-up participants did not differ significantly from the initial sample.

- Please clarify that Pearson correlation primarily reflects rank-order stability. Reporting an ICC with a confidence interval would provide a more complete assessment of test–retest reliability, if feasible.

**Response**: We thank the Reviewer for these five points, each of which we have addressed.

Number of predictors in the power analysis. The Reviewer is correct that the regression models include the SCS together with four demographic control variables. The G*Power calculation has been corrected accordingly, and the required sample size revised from 89 to 95. The achieved follow-up sample of 97 participants remains above this threshold, so the recruitment targets reported in the manuscript are unaffected. The passage now reads:

With an effect size of .19, a significance level of 0.05, a power of .95, and five predictors (SCS and four demographics), at least 95 individuals were needed.

Notation for statistical power. We have corrected the notation in both studies, so that power is now reported as 1 − β = .95 rather than β = .95. In Study 2 the sentence now reads: "Sensitivity analysis, assuming α = .05 and 1 − β = .95, yielded critical values of .04 for regression analyses and .08 for correlation analyses."

Actual follow-up interval. The Reviewer is right that the interval was not exactly seven weeks for every participant. Because the first wave was administered over a five-week recruitment window while the second wave was administered within a single week, the realised interval varied. We have amended the sample size rationale to describe seven weeks as the planned rather than the realised interval, and have added the observed values to the Participants section:

Because the first wave was administered over a five-week recruitment window while the second wave was administered within a single week, the interval between measurements varied across participants, ranging from 6.86 to 11.14 weeks (M = 8.43, SD = 1.54).

We have also revised the two statements in the Discussion that referred to a seven-week timeframe so that they reflect the interval actually observed.

Statistical details for the attrition comparison. We have replaced the unsupported statement with the descriptive characteristics of the follow-up subsample and the corresponding tests:

The follow-up subsample consisted predominantly of women (70.1%), with a mean age of 31.4 years (SD = 8.8) and 8.4 years of work experience (SD = 7.8). Participants who completed the follow-up (n = 97) did not differ significantly from those who did not (n = 358) in gender, χ²(1) = 0.11, p = .745, age, t(453) = 0.91, p = .363, or work experience, t(453) = −0.67, p = .506.

Pearson correlation and the intraclass correlation coefficient. We agree, and have added the ICC as suggested. The Results now read:

The scores from two time points for each participant were correlated, yielding a significant (p < .001) and high correlation coefficient (r = .81). As the Pearson coefficient reflects rank-order stability and is insensitive to systematic mean-level change between measurements, absolute agreement was additionally assessed using a two-way random-effects intraclass correlation coefficient for single measurements (Shrout & Fleiss, 1979), ICC(2,1) = .80, 95% CI [.72, .86]. The close correspondence between the two coefficients indicates that the stability of SCS scores was not accompanied by systematic mean-level change.

Shrout and Fleiss (1979) has been added to the reference list.

**Comment 1.5.** 5. Cross-cultural claims: The revised Conclusions are appropriately cautious, but some statements in the Abstract remain too strong, particularly the claims of “robust empirical evidence” and validation “across cultural contexts.”

The findings would be more accurately described as providing preliminary evidence supporting the Polish adaptation of the SCS in the present samples. The authors may also consider revising the title to emphasize “Polish Adaptation and Validation.”

**Response**: We thank the Reviewer for this observation and agree that the Abstract had not been brought fully into line with the revised Conclusions. We have moderated the Abstract, revised the title as the Reviewer suggests, and adjusted the keywords for consistency.

The two claims identified by the Reviewer have been removed. The closing statements of the Abstract previously read: "This study provides robust empirical evidence supporting the theoretical model of sustainable careers, confirming the distinctiveness and coherence of its four dimensions. The findings validate the model's applicability across cultural contexts and underscore the significance of sustainable career strategies." They now read:

This study provides preliminary evidence supporting the Polish adaptation of the SCS in the present samples, confirming the distinctiveness of its four dimensions. The findings support the applicability of the sustainable careers model in the Polish context and underscore the significance of sustainable career strategies.

In addition, we revised the description of the psychometric results so that the Abstract reflects the qualified conclusions now stated in the Results and Discussion, including the limitations concerning the Health dimension addressed under Comment 3. The sentence previously claiming "high internal consistency, reliability, and strong validity" now reads:

The Polish adaptation of the SCS maintained its four-factor structure (happiness, health, productivity, and social empowerment) and demonstrated high internal consistency and temporal stability, along with criterion and discriminant validity evidence and lagged associations with well-being outcomes, with some limitations for the Health dimension.

Following the Reviewer's suggestion, the title has been changed from "Cross-Cultural Validation of the Sustainable Career Scale in the Polish Context" to:

Polish Adaptation and Validation of the Sustainable Career Scale

For consistency with the revised title, the keyword "cross-cultural validation" has been replaced with "scale adaptation." The title has been updated in the manuscript file and the submission system.

**Comment 1.6. 6**. Sample independence: The response letter states that Study 1 and Study 2 used independent samples with no participant overlap. Please include this clarification explicitly in the manuscript and briefly explain how duplicate participation across the two studies was prevented or assessed.

**Response**: We thank the Reviewer for this request. We have added the clarification to the manuscript, in the Participants and Sampling section of Study 2:

Study 1 and Study 2 were administered with LimeSurvey's cookie-based duplicate-response control enabled, so that repeated participation across the two studies was prevented as far as the platform allows.

The duplicate-response control was configured to apply across both studies (by manually altering Study 2 id within Lime Survey platform) rather than within each separately. We took this measure to ensure that the samples would be independent, and have phrased the statement accordingly.

**Comment 1.7. 7**. Data availability and presentation: Please clarify whether the analysis scripts are publicly available in addition to the raw data, processed data, codebook, Polish scale, and scoring instructions.

**Response**: We thank the Reviewer for raising this. In addition to the existing shared data, we have now also deposited the R scripts used for data processing and analysis at the same OSF repository. The Data Availability Statement has been updated accordingly:

Data availability statement. The anonymized raw and processed data, the codebook, the full Polish version of the developed scale with scoring instructions, and the R scripts used for data processing and analysis are openly available at the Open Science Framework (https://osf.io/2n4r5/files/).

We note that these scripts were originally written for internal use over the course of the project and were not prepared with external readability in mind; we have therefore included a brief note in the repository inviting researchers who wish to reproduce a specific analysis to contact the author responsible for the statistical work directly, so that any questions about the scripts can be addressed.

**Comment 1.8.** Clarify whether the second set of factor loadings in Table 1 represents EFA or CFA results.

**Response**: We thank the Reviewer for identifying this ambiguity. The second set of loadings in Table 1 (Study 1) is from the confirmatory factor analysis of the four-factor correlated model, not from an exploratory factor analysis; only the Pilot column reflects the EFA. The table note previously attributed both columns to the exploratory factor analysis, which was incorrect. It has been corrected to read:

loadings = factor loadings for the corresponding factor per item, obtained from an exploratory factor analysis for the Pilot and from the confirmatory factor analysis of the four-factor correlated model for Study 1.

**Comment 1.9.** Add the full reference for Robinson (2017), if retained.

**Response**: We thank the Reviewer for catching this omission. The full reference for Robinson (2017) has been added to the reference list:

Robinson, S. R. (2017). Engaging a multigenerational workforce [Doctoral dissertation, Walden University]. ScholarWorks. https://scholarworks.waldenu.edu/dissertations/4094

**Comment 1.10**. Clarify whether the descriptive statistics for the negatively worded Health items were calculated before or after reverse scoring.

**Response**: We thank the Reviewer for this question. The descriptive statistics for the Health items in Table 1 (items 4–6) are calculated prior to reverse scoring, on the original item wording; reverse scoring is applied when computing the Health subscale total and in all subsequent substantive analyses. We had previously described the items as reverse-scored "prior to all analyses," which did not accurately reflect Table 1, and have corrected this in the Measurements section and added a clarifying note to Table 1 directly:

Measurements: "The three health items (items 4–6), which are negatively worded, were reverse-scored prior to the calculation of scale totals and all substantive analyses, so that higher scores indicate a higher level of the respective dimension. Item-level descriptive statistics reported in Table 1 are presented on the original, non-reversed item scale."

Table 1 note: "...Descriptive statistics for items 4–6 (Health) are reported prior to reverse scoring."

**Comment 1.11**. Use the terms criterion, convergent, discriminant, and divergent validity consistently.

**Response:** We thank the Reviewer for this observation. We identified and corrected two inconsistencies in validity terminology.

First, one summary passage referred to the SCS's "criterion-related, divergent, and prospective aspects," where "divergent" should have read "discriminant," consistent with Hypothesis 5 and the corresponding Results. This has been corrected.

Second, "criterion validity" (used in Hypothesis 4 and its Results) and "criterion-related validity" (used in three summary passages) were used interchangeably to refer to the same construct. We have standardized these to "criterion validity" throughout.

**Comment 1.12**. Check the Polish wording of Item 10.

**Response**: We thank the Reviewer for catching this. This has been corrected to "budowania."

**Comment 1.13**. Correct the remaining grammatical, punctuation, spacing, and formatting errors.

**Response**: We thank the Reviewer for this. We have proofread the manuscript and corrected the remaining errors. These included several grammatical constructions (for example, "for correctness and natural sounding," now "for correctness and naturalness"), one incomplete clause in the Hypothesis 1 results, inconsistencies in the reporting of alpha and power notation, and remaining punctuation and spacing errors, including double periods and orphaned punctuation left by earlier deletions. Citation formatting has also been corrected where needed.
