# Supplementary material for: Polish adaptation and validation of the sustainable career scale
Source: PLoS One. 2026 Sep 8;21(9):e0357496. doi: 10.1371/journal.pone.0357496 (PMC13552746; doi:10.1371/journal.pone.0357496)
Supplement: Supplementary file 4 [file pone.0357496.s002.docx]

# **Responses to Reviewers**

**Reviewer #1:** Thank you for the opportunity to review this manuscript, entitled 'Cross-Cultural Validation of the Sustainable Career Scale in the Polish Context'. The study addresses a relevant topic in career psychology and makes a potentially useful contribution by adapting and validating the Sustainable Career Scale in the Polish context. The multi-stage design, including qualitative assessment, pilot testing, confirmatory factor analysis, validity testing, and follow-up data, is appropriate in principle for a cross-cultural validation study.
However, in its current form, the manuscript requires substantial revision before it can be considered technically sound for publication. My main concerns relate to the clarity of the study contribution, the independence of the samples, the measurement invariance analysis, statistical reporting, interpretation of the hierarchical model, predictive validity claims, ethics reporting, and the strength of the conclusions.

**Author’s respond**: We thank Reviewer for the thorough and constructive evaluation of the manuscript. The comments identified several important issues that have substantially strengthened the paper, and we have addressed each point in full. Below we respond to each comment in turn, with the corresponding revisions indicated.

| **Reviewer’s comment** | **Response** |
| --- | --- |
| 1. The contribution of the study should be framed more cautiously. The manuscript provides new evidence for the Polish adaptation of the Sustainable Career Scale, which is valuable. However, the original scale has already been developed and validated in previous research, including evidence on factor structure, reliability, validity, predictive validity, and gender invariance. Therefore, the present contribution should be described more specifically as a Polish cultural adaptation and validation study, rather than as broad evidence for the universal cross-cultural applicability of the scale. | We thank the Reviewer for this comment. We agree that the original framing overstated the contribution relative to what a single Polish sample can establish, particularly given that the original scale has already been validated for factor structure, reliability, validity, predictive validity, and gender invariance. We have revised the contribution statement in the Introduction and the opening of the Theoretical Contribution section to frame the study specifically as a Polish cultural adaptation and validation, extending the existing evidence to a new cultural context rather than claiming broad cross-cultural applicability. The revised text reads as follows:  Introduction (updated part):  *Secondly, it offers empirical support for Russo et al.'s innovative theoretical model of career sustainability by adapting and validating the scale within a new cultural context. As the original scale has already been validated for factor structure, reliability, validity, predictive validity, and gender invariance (Russo et al., 2025), the present contribution lies in extending this evidence to the Polish cultural context, providing an initial indication that the model's core dimensions are recognisable beyond the setting in which it was originally developed.*  Discussion (opening sentence updated):  *This research advances the field of career psychology by providing evidence for the Polish cultural adaptation and validation of the SCS, demonstrating that the core dimensions of career sustainability—happiness, health, productivity, and social empowerment—are recognisable and measurable within a new cultural context.* |
| 2. The independence of Study 1 and Study 2 needs clarification. A major concern is that Study 1 and Study 2 appear to report very similar recruitment numbers and overlapping data collection periods. For example, both studies appear to report that 654 individuals started the survey. The authors should clearly explain whether Study 1 and Study 2 used fully independent samples, partially overlapping samples, or different analyses from the same recruitment wave. If the samples overlap, the manuscript should not present them as independent validation studies. | We thank the Reviewer for raising this, and we apologise for the confusion. Study 1 and Study 2 used fully independent samples with no participant overlap. The reported figure of 654 starting participants for Study 2 was a typographical error introduced during the manuscript's previous rounds of revisions. The correct initial number for Study 2 was 633, which we have corrected in the text. To make the independence of the samples explicit, we have added a statement to the Introduction clarifying that the two studies were conducted on separate, independent samples. We regret that this error persisted across the manuscript's several rounds of revision, and we have re-checked the recruitment figures throughout to ensure consistency. |
| 3. The measurement invariance analysis should be revised. The manuscript states that measurement invariance was examined across gender and age groups. However, the current reporting seems to rely mainly on separate CFA models for different groups rather than formal multi-group measurement invariance testing. A proper invariance analysis should test configural, metric, scalar, and, where appropriate, residual invariance, using established criteria such as changes in CFI, RMSEA, and SRMR. Without such testing, the claim that the scale is invariant across gender or age groups is not sufficiently supported. | We thank the Reviewer for this important comment. We agree that the original approach did not constitute formal invariance testing. We have replaced it with a multigroup measurement invariance analysis, testing configural, metric, scalar, and residual invariance across gender and age. Following established recommendations, invariance was judged primarily by changes in approximate fit indices (ΔCFI ≤ .010, ΔRMSEA ≤ .015, ΔSRMR ≤ .030/.010; Chen, 2007; Cheung & Rensvold, 2002; Meade et al., 2008), with the χ² difference test reported but not treated as decisive given its sample-size sensitivity. Full residual invariance was supported across gender, and invariance held across age between Generations Z and Y. Generation X was excluded from this analysis, as the subgroup (n = 65) was too small for stable multigroup estimation. The revised procedure and results are reported in the Data Analysis and Results sections and Table 3, and the Abstract and Discussion were updated accordingly.  Results:  *Measurement invariance was tested using a multigroup approach, with the four-factor correlated model fitted under progressively more restrictive equality constraints (configural, metric, scalar, and residual). Because the χ² difference test is a known function of sample size and tends to flag trivial differences as significant in larger samples (Bentler & Bonett, 1980; Cheung & Rensvold, 2002), invariance decisions were based primarily on changes in approximate fit indices, with ΔCFI ≤ .010, ΔRMSEA ≤ .015, and ΔSRMR ≤ .030 (loadings) or ≤ .010 (intercepts and residuals) taken to indicate invariance (Chen, 2007; Meade et al., 2008); the χ² difference test is nonetheless reported for transparency. Full results are presented in Table 3.*  *Invariance across gender (female n = 352, male n = 131; 14 cases were excluded as gender was not reported) was supported at every level. The configural model fitted the data well (CFI = .954, RMSEA = .075, SRMR = .055), and imposing equality of loadings (metric), intercepts (scalar), and residuals produced negligible changes in fit at each step (all ΔCFI ≤ .006, all ΔRMSEA ≤ .003, all ΔSRMR ≤ .001), well within the adopted thresholds; the χ² difference tests were correspondingly non-significant (metric Δχ²(8) = 1.50, p = .993; scalar Δχ²(8) = 10.40, p = .240; residual Δχ²(12) = 4.47, p = .973). Strict residual invariance was therefore established across gender. Invariance across age was tested between Generation Z (n = 269) and Generation Y (n = 163); Generation X (n = 65) was excluded, as the subgroup was too small for stable multigroup estimation. The configural model again fitted well (CFI = .965, RMSEA = .064, SRMR = .055), and all subsequent constraints fell within the adopted fit-difference criteria (largest ΔCFI = −.007 at the scalar step; all ΔRMSEA ≤ .003; all ΔSRMR ≤ .006). The χ² difference tests were significant at the metric (Δχ²(8) = 16.97, p = .031) and scalar (Δχ²(8) = 24.60, p = .002) steps; however, consistent with the rationale above, these were not treated as decisive, as the accompanying changes in CFI, RMSEA, and SRMR indicated no meaningful deterioration in fit (Chen, 2007; Cheung & Rensvold, 2002). Residual invariance was likewise supported (Δχ²(12) = 6.31, p = .900). Taken together, these results support Hypothesis 2: the Polish version of SCS demonstrated measurement invariance across both gender and age groups, allowing meaningful comparison, with the qualification that age invariance was established for Generations Z and Y only.*  Discussion (updated paragraph):  *Measurement invariance held across gender, and full invariance was also supported between Generations Z and Y, indicating that the SCS functions equivalently for these groups. Invariance could not be tested for Generation X, as the subgroup was too small for stable estimation; this limitation may be relevant given that generational cohorts can differ in the cultural values they emphasise, such as hedonism, traditionalism, and universalism (Ismail, 2016). Generation X has been characterised as prioritising traditional career paths and placing less emphasis on work-life balance (Jorgensen, 2003; Otken & Erben, 2013). Whether such generational differences affect the measurement properties of the scale across older cohorts remains an open question, particularly as younger generations tend to favour non-linear careers and show greater sensitivity to social and environmental impact (Lyons et al., 2012; Zemke et al., 1999).* |
| 4. Several statistical reporting issues require correction. There are inconsistencies in the reported sample sizes. For example, the subgroup sample sizes in the invariance table do not appear to align clearly with the final sample size reported for Study 1. The reported age-group sample size for Generation X also appears problematic given the total sample size. In addition, the Bartlett test reported in the pilot study seems to include a degree of freedom value that may not correspond to a 12-item factor analysis. The authors should carefully re-check all statistical values, sample sizes, degrees of freedom, and table entries. | We thank the Reviewer for this careful reading, which prompted a thorough re-check of all statistical values, sample sizes, and degrees of freedom.  We revised all information on sample sizes, as described in our response to Comment 2. The Generation X issue stemmed from a tracked-changes formatting error. The invariance subgroup sizes now reconcile with the Study 1 sample. The reported Bartlett value (df = 11) had been produced by Bartlett's test of homogeneity of variances rather than Bartlett's test of sphericity required for factorability. We re-ran the correct test of sphericity on the 12-item correlation matrix; it confirms factorability, now correctly reported as χ²(66) = 1053.87, p < .001.  As noted in our response to a further comment, the CFA indices were also revised, as the models were re-run with a different estimator (MLR). Finally, we re-checked all remaining values across the text and tables and corrected several minor inconsistencies, including the information criteria reported in the text to match Table 3, the square-root-of-AVE values, and minor reporting and typographical errors. |
| 5. The claim that the hierarchical four-factor model is superior is not fully supported.  The manuscript argues in favour of the hierarchical four-factor model. However, the reported fit indices seem to suggest that the four correlated-factor model may fit the data better on some criteria. The authors should provide a clearer comparison of competing models, including fit indices and, where appropriate, AIC, BIC, and chi-square difference tests. The justification for using a total SCS score should also be reconsidered and discussed more cautiously. | We are grateful for this comment, which prompted us to re-examine a conclusion we had not stated with sufficient care. The Reviewer is correct: on closer inspection of the fit indices, the four correlated-factor model fits at least as well as, and on most criteria better than, the hierarchical model, and our original wording overstated the support for the higher-order specification.  To adjudicate between the two more rigorously, we supplemented the fit indices with the AIC and BIC. To do this, we also re-run entire CFA analysis with MLR estimator, as the previously used WLMSV would not allowed for calculating these metrics, and that woud create a discrepancies between previous fit indices and newly added.  As a results, both four-factor solutions decisively outperformed the single- and three-factor models, confirming the four-factor structure; between them, the correlated model held a modest advantage. Because the two remained close, we then compared them directly with a scaled chi-square difference test. The hierarchical model fit significantly worse than the correlated one, so the information criteria and the difference test converge on the same conclusion.  We have revised the manuscript throughout to reflect this. Hypothesis 1 is now framed as not supported. Although the four-factor conceptualization is strongly confirmed, the theoretically anticipated hierarchical structure is not favoured over four correlated factors. As such, the Results and Discussion have been rewritten accordingly, with the additional indices reported in Table 3.  Following the Reviewer's recommendation, we also reconsidered the use of a total SCS score, which the Discussion now treats more cautiously: it is endorsed only as a coarse summary index (e.g., for screening or as a control variable), while dimension-level questions are directed to the four subscale scores.  The updated text now reads as follows:  Results:  *Because the hierarchical and correlated four-factor models were closely matched on the primary fit indices, a further set of comparisons was conducted to adjudicate between them. The models were first compared using the Akaike (AIC) and Bayesian (BIC) information criteria. Consistent with the primary results, both four-factor solutions produced substantially lower AIC and BIC values than the single-factor solution (AIC = 20006.09, BIC = 20107.05) and the three-factor solutions (lowest three-factor AIC = 18712.08, BIC = 18825.65), confirming the superiority of the four-factor structure. The two four-factor models nonetheless remained close, with the correlated model showing a slight advantage on both criteria (AIC = 17938.60, BIC = 18064.80) over the hierarchical model (AIC = 17966.10, BIC = 18083.89).*  *Given this proximity, the two nested models were compared directly using a scaled chi-square difference test (Satorra, 2000), which is appropriate for a robust estimator and computes the difference statistic from the standard, rather than the scaled, per-model test statistics. The test indicated that the hierarchical model fit the data significantly worse than the correlated model, Δχ²(2) = 34.63, p < .001. Thus, although the hierarchical model met conventional thresholds for acceptable fit in absolute terms, imposing the second-order structure produced a significant loss of fit relative to the correlated solution. The information criteria and the scaled difference test converge on the same conclusion: the four-factor structure is strongly supported, but the data favor a model of four correlated factors over the theoretically anticipated hierarchical one. Consequently, interpreting the four factors as correlated dimensions is empirically preferred, while a general score remains defensible on parsimony grounds. Hypothesis 1 is therefore not supported, as the four-factor conceptualization of career sustainability is clearly upheld, whereas the superiority of the higher-order specification is not.*  Discussion:  *The career sustainability model advanced by Russo et al. (2023, 2025) holds that happiness, health, productivity, and social empowerment are not merely co-occurring outcomes but converge on a single overarching construct, echoing De Vos et al.'s (2020) conception of sustainable careers as an integrated person-level state rather than a loose bundle of indicators. This theoretical premise motivated Hypothesis 1 and its expectation of a hierarchical structure in which one general factor underlies the four dimensions. The data only partly bear this out. That the four dimensions are empirically distinct yet related emerged as the clearest and most robust result, affirming the multidimensional core of the model. Their reduction to a common higher-order factor, however, found weaker support: the hierarchical specification fit less well than one of four correlated factors. This suggests that the dimensions are more plausibly understood as a family of related but autonomous facets than as expressions of a single underlying construct. Such a reading aligns with conceptualizations that treat career sustainability not as one latent trait but as an emergent property arising from the interplay of distinct components—health, happiness, productivity, and social empowerment—each shaped by different personal and contextual forces and capable of developing, or eroding, on its own (De Vos et al., 2020; Van der Heijden & De Vos, 2015). If the dimensions indeed operate as semi-autonomous facets rather than indicators of one trait, a total SCS score should be treated with corresponding caution: it can serve as a rough summary where only a single, global indication of career sustainability is required, e.g., in brief screening or as a control variable, but it inevitably masks the divergent ways in which the facets may relate to their antecedents and outcomes. Wherever those differences are of interest, the four subscale scores should be retained rather than aggregated.* |
| 6. The EFA reporting in the pilot study is insufficient. The exploratory factor analysis is reported rather briefly. The authors should provide a fuller factor loading matrix, including cross-loadings, eigenvalues, factor correlations, and communalities. Reporting only selected or target-factor loadings does not allow readers to evaluate the factorial clarity of the adapted scale. | We thank the Reviewer for this helpful comment. We fully agree that the original reporting was too brief to allow a proper evaluation of the adapted scale's factorial clarity, and we have substantially expanded it. The complete pattern matrix is now provided, including all factor loadings and cross-loadings, together with the communalities, the eigenvalues of the correlation matrix, a parallel analysis for factor retention, and the inter-factor correlations. Each of these is reported in full and accompanied by an interpretation.  At the same time, we remain mindful that the pilot EFA was preliminary and exploratory in nature and was conducted on a small sample. We therefore do not regard these analyses as providing decisive evidence on their own, and we were concerned that presenting them at length within the main text might lead readers to interpret them as more conclusive than they are warranted to be. For this reason, and to avoid further expanding an already lengthy manuscript, we have moved the expanded EFA reporting to Appendix A, where it is presented and explained in full, while the main text retains a concise summary with a clear reference to the appendix. We hope the Reviewer finds this approach, and the level of detail now provided, sufficient. |
| 7. The interpretation of AVE and discriminant validity needs clarification. The manuscript appears to use AVE, the square root of AVE, and 'squared AVE' inconsistently. The authors should clarify exactly what is reported in the relevant tables and ensure that the Fornell-Larcker criterion is applied correctly. If the diagonal values in the correlation tables are AVE values rather than square roots of AVE, the interpretation should be revised. | We thank the Reviewer for catching this inconsistency. The term "squared AVE" was a writing error: the values reported on the diagonals of the relevant tables, and used in the discriminant validity assessment, are the square roots of AVE throughout, as required by the Fornell-Larcker criterion. The underlying values and analyses were correct; only the terminology was inconsistent. We have corrected all instances in the text to "square root of AVE" and clarified the table notes to state explicitly that the diagonal values represent the square root of AVE. |
| 8. The predictive validity evidence should be interpreted more cautiously. Study 2 is presented as evidence of predictive validity. However, the follow-up sample is relatively small, and the analyses do not appear to control for baseline levels of the outcome variables. Therefore, the findings may be better described as lagged associations rather than strong evidence of predictive validity. If regression assumptions such as homoscedasticity or normality of residuals are not fully met, the authors should conduct appropriate robustness checks or soften the conclusions. | We thank the Reviewer for this comment, which we have addressed in two ways. First, as the follow-up sample did not include a baseline measure of the outcomes, we agree the findings are better characterised as lagged associations rather than predictive validity in the strict sense, and we have revised the relevant hypothesis, analysis description, and results throughout the manuscript to reflect this framing. Second, regarding the regression assumption violations in the Life Satisfaction model, we re-estimated the model using HC3 heteroskedasticity-consistent standard errors to address the heteroskedasticity, and bootstrapped the coefficient confidence intervals (2,000 resamples) to avoid reliance on the normality assumption. The career sustainability coefficient remained significant and of comparable magnitude under both checks, confirming the original result was not an artifact of the identified violations and allowing us to confirm support for Hypothesis 6. These robustness checks are now reported alongside the original estimates in Table 5.  The updated Results section now reads as follows:  *However, several issues were identified with the Life Satisfaction model, notably signs of heteroskedasticity (GQ = 2.11, p < .01) and deviations from normality in the residuals (W = .96, p < .05). To investigate the underlying causes, the data were analyzed for potential outliers. Neither Studentized residuals (>3) nor Cook's distances (>1) indicated the presence of outliers. To formally address these violations, the model was re-estimated using HC3 heteroskedasticity-consistent standard errors, and coefficient confidence intervals were additionally bootstrapped (2,000 resamples) to avoid reliance on the normality assumption, as reported in Table 5. The career sustainability coefficient remained significant and of comparable magnitude under both checks (b = .79, HC3 p < .001, bootstrap 95% CI [.55, 1.02]), closely matching the original estimate and confidence interval. As the robustness checks confirmed the original result was not an artifact of the identified violations, the presented data support Hypothesis 6.* |
| 9. The ethics statement is internally inconsistent. The submission information states that formal ethics approval was not required, whereas the manuscript states that approval was obtained from the ethics committee of Adam Mickiewicz University. This inconsistency must be resolved. The authors should clearly state whether the study received formal ethics approval, was granted an exemption, or did not require review under institutional rules. If approval was obtained, the name of the ethics committee, approval number, and approval date should be provided. The authors should also clarify how email addresses collected for the follow-up study were stored, separated from survey responses, and deleted or anonymised. | We thank the Reviewer for raising this, and we apologise for the inconsistency. The discrepancy arose from the timing of the submission rather than from any ambiguity about the study's ethical status. The study did receive formal ethics approval from The Ethics Committee for Research Projects at Adam Mickiewicz University, Poznań (approval number 07/03/2026; issued 17/03/2026). The conflicting statement in the submission information predated the finalisation of this record; the manuscript was subsequently updated during the technical check process to reflect the correct and current position. The version in the manuscript is therefore the accurate one, and we have ensured that the ethics statement is now internally consistent and complete, specifying the approving committee, the approval number, and the approval date.  Regarding the follow-up procedure, we have added a clarification to the Method section. Participants' email addresses were managed using the token feature of LimeSurvey: they were stored in a separate file on an internal university server, kept apart from the survey responses, and used only to issue the follow-up invitation that involves unique token code. Responses from the two waves were linked via a unique token code rather than the email address, and all email addresses were deleted immediately after the data were merged. No identifying information is retained in the archived dataset.  The updated text reads as follows:  *To link the two waves while protecting privacy, we used LimeSurvey's token feature. The survey dataset contained each participant's unique token but no email address; a separate file, stored on an internal university server, held the token–email pairings used to send invitations. The two waves were merged via the token, and the separate file with email addresses was deleted immediately afterwards.* |
| 10. The conclusions should be more closely aligned with the evidence. The findings support the reliability and validity of the Polish version of the SCS in the present samples. However, they do not justify broad claims about universal cross-cultural applicability across diverse cultural settings. The conclusion should be revised to state more cautiously that the study provides preliminary evidence for the Polish adaptation of the SCS in Polish worker samples, and that further research with independent, representative, and occupationally diverse samples is needed. | We thank the Reviewer for this helpful suggestion, which we have adopted in full. We have revised the Conclusions so that the contribution is framed as preliminary evidence for the Polish adaptation of the SCS in Polish worker samples, replacing the earlier references to "successful validation" and "cross-cultural applicability." The statement on the dimensions' broader relevance has been tempered to indicate that the model's applicability may extend beyond its original context, and we have added an explicit call for further research with independent, representative, and occupationally diverse samples. For consistency, we have applied the same more measured framing to the Abstract and Discussion. |
| 1. The terminology should be standardised throughout the manuscript. In particular, the manuscript appears to alternate between 'social empowerment' and 'social impact'. One term should be selected and used consistently. | We thank the Reviewer for noting this. We have standardised the terminology to "social empowerment" throughout the manuscript, including all tables. |
| 2. The use of GPT-3.5 in the translation process should be described more transparently. The authors should clarify its exact role, how the AI-generated output was checked by human experts, and how conceptual equivalence was ensured. | We thank the Reviewer for this comment and have expanded the relevant passage to make the procedure clearer. We should clarify that the GPT-3.5 output functioned only as supplementary material supporting a fully human translation process. The GPT-3.5 translation itself was generated following the approach we examined in Grobelny et al. (2025), using our previously tested prompt and resources for supporting the model; the resulting translated items were then made available to the two psychologists as additional reference material during their discussion. The translation and reconciliation themselves were carried out entirely by the human translators. This was therefore not a human-in-the-loop procedure in which the model performs translation under human oversight, but a fully human approach in which the AI-generated items served only as an auxiliary reference. For this reason, no test of equivalence between an AI version and a human version was conducted: the GPT-generated material was never an independent translation, but a resource that supported the two researchers in reaching a collective decision. This is also why we had not originally emphasised the AI component or cited the underlying method; we did not wish to overstate its role. We have nonetheless updated the text to describe the procedure transparently, with the focus kept on how the human translators carried out and reconciled the translation.  Grobelny, J., Szymański, K., & Strozyk, Z. (2025). Act as an expert in psychometry: The evaluation of large language models' utility in psychological tests' cross-cultural adaptations. Acta Psychologica, 261, 105813. <https://doi.org/10.1016/j.actpsy.2025.105813>  As a result, this text:  *The translations were then compared, and any significant discrepancies were resolved through discussions, utilizing the AI-generated translations as an auxiliary source of information*  Has been replaced with this:  *The two psychologists' translations were then compared during a joint session. Both were additionally provided with the GPT-3.5 translation, which they evaluated by comparing its differences against their own versions. Through discussion, they integrated their two translations into a single version, resolving any discrepancies collectively, with the GPT-3.5 version available to them only as supplementary, informative material rather than as an authoritative source* |
| 3. The focus group component is underreported. More detail is needed on the participants, coding procedure, interpretation of responses, and how the qualitative findings informed item revision. | We thank the Reviewer for this comment. We agree that the original reporting of the focus group component was too brief to allow a proper evaluation of its contribution to the adaptation process. We have substantially expanded this section, moving the detailed FGI reporting to Appendix A to preserve space in the main manuscript. The appendix now provides a full item-level comprehension table (Table A1) reporting, for each item, the number of participants who rated it as clear, expressed uncertainty, and reported knowing how to respond, alongside representative participant quotes organized by two coding categories: item comprehension (difficulties related to wording, scope, or item referent) and construct understanding (broader reflections on the expected content of the questionnaire and participants' lay definitions of sustainable career). A narrative summary accompanying the table describes comprehension patterns across subscales, noting that Happiness and Productivity items received consistently high ratings while Social Empowerment items generated the most uncertainty, particularly regarding their occupational relevance and overlap between items. These qualitative findings directly informed the decision to retain all items while proceeding with particular caution regarding the Social Empowerment subscale, which was subsequently subjected to additional quantitative scrutiny in the primary studies. The main text retains a concise summary with a clear reference to the appendix. |
| 4. The manuscript contains several grammatical errors and repeated sentences. Careful language editing is recommended. | We thank the reviewer for this comment. We reviewed the manuscript to address grammatical issues and repetitions. We hope that the revised version is now clear, concise, and free of language-related problems. |
| 5. Some table labels should be checked. For example, if 'alpha when dropped' reports negative values or changes in alpha, the table heading should be revised to avoid misunderstanding. | We thank the Reviewer for catching this. The column did report the change in alpha rather than the resulting alpha, and the original heading was misleading.  We have relabelled it "Δα" and added a note to the table: "Δα = change in Cronbach's alpha if the item were removed; negative values indicate that removing the item would reduce scale reliability." |
| 6. The scoring of negatively worded health items should be explained more clearly, including whether reverse coding was applied before reliability, CFA, and validity analyses | We thank the Reviewer for raising this. The negatively worded health items (items 4–6) were reverse-scored prior to analyses (reliability, factor analyses, and validity), so that higher scores consistently indicate a higher level of the respective dimension.  We have now clarified it in the Measurements section where the SCS is first described, specifying which items were reverse-scored and that this was done before any analyses were conducted.  The text reads as follows:  *The three health items (items 4–6), which are negatively worded, were reverse-scored prior to all analyses, so that higher scores indicate a higher level of the respective dimension.* |
| 7. The authors should specify exactly what is available on OSF, including anonymised raw data, processed data, codebook, Polish scale items, scoring instructions, and analysis scripts. | We thank the Reviewer for this suggestion. We have revised the Data Availability Statement to specify the OSF contents: the anonymised raw and processed data, the codebook, and the full Polish scale with scoring instructions.  The statement now reads as follows:  *Data availability statement. The anonymised raw and processed data, with the codebook, and the full Polish version of the developed scale, with scoring instructions, are openly available at the Open Science Framework (https://osf.io/2n4r5/files/).* |
| 8. The preregistration statement is too brief. The authors should clarify which analyses were preregistered and which analyses, if any, were exploratory. | We thank the Reviewer for this suggestion. We have expanded the preregistration statement to specify which analyses were preregistered and which were exploratory. All core analyses were conducted as registered; the AIC/BIC and scaled chi-square comparison of the two four-factor models, the parallel analysis, and the expanded factor-structure reporting in Appendix A are now explicitly labelled as exploratory additions.  The added statement part now reads as follows:  *The core analyses were conducted as planned: the pilot EFA and reliability analyses, the focus group interviews; in the primary studies, the descriptive statistics, power/sensitivity analyses, reliability estimates, the confirmatory factor analysis comparing the competing models, measurement invariance, the AVE-based discriminant validity assessment, common method bias estimation, the criterion-validity correlations, the predictive-validity regressions (here as lagged associations), and test–retest reliability. Three analyses were not part of the registered protocol: the comparison of models using AIC, BIC, a scaled chi-square difference test, the parallel analysis and expanded factor-structure reporting in Appendix A, all added during revision.* |

**Reviewer #2:** Thank you for giving me the opportunity to review this manuscript. The overall research design is relatively rigorous, with well-established and appropriate statistical methods, clear empirical logic, and reasonable and orderly argumentation. It has certain theoretical and practical research value in the field of occupational psychology. However, there are also several issues that can be discussed, such as:

**Author’s respond**: We thank Reviewer for the careful reading of the manuscript and for the constructive comments. Below we address each point in turn, indicating the corresponding revisions made to the manuscript. A summary table of all comments and our responses is provided below for ease of reference.

| **Reviewer’s comment** | **Response** |
| --- | --- |
| 1.The content of lines 7-13 on page 7 is repetitive, and it is recommended to revise it. | We thank the Reviewer for catching this. As the passage contained a duplicated pair of sentences, we have removed the repetition. |
| 2.In all three samples (pilot, Study 1, Study 2), the proportion of females exceeds 70%, the proportion of white-collar workers/service industry employees exceeds 70%, and the proportion of individuals with higher education exceeds 70%. It is evident that there are certain limitations in the representativeness of the samples. It is recommended to point this out more explicitly in the "Limitations" section. | We thank the Reviewer for this comment. The Limitations section previously addressed only the white-collar concentration of the sample; we have expanded it to explicitly note the gender and education skew as well, and to point to its consequences for the productivity and social empowerment dimensions specifically. The Limitations section now reads as follows:  *Across all three samples, women, service-sector and white-collar employees, and individuals with higher education were each represented at over 70%, limiting the generalizability of the findings. This homogeneity is particularly relevant for the productivity and social empowerment dimensions, which may carry different meaning for blue-collar or lower-autonomy workers, potentially affecting both the relevance of specific items and the observed factor structure in more occupationally diverse populations.* |
| 3.The sample size of Generation X is below the minimum recommended sample size for CFA, leading to poor model fit. The author acknowledges this limitation but still concludes that "the data support H2". | We thank the Reviewer for this comment. We have substantially revised our approach to age-based measurement invariance in response to this and a related comment from Reviewer 1. Rather than relying on separate per-group CFAs, we now conduct formal multigroup invariance testing (configural, metric, scalar, residual). The Generation X subgroup (n = 65), which is indeed below the recommended minimum for stable CFA estimation, was therefore excluded from the invariance analysis rather than retained with poor model fit; invariance was tested between Generations Z and Y only, and the conclusion regarding Hypothesis 2 is now scoped accordingly rather than claiming support across all age groups. The revised text reads as follows:  *Invariance across age was tested between Generation Z (n = 269) and Generation Y (n = 163); Generation X (n = 65) was excluded, as the subgroup was too small for stable multigroup estimation. […] Taken together, these results support Hypothesis 2: the Polish version of SCS demonstrated measurement invariance across both gender and age groups, allowing meaningful comparison, with the qualification that age invariance was established for Generations Z and Y only.* |
| Additionally, is there an error in the sample size presented in Table 3? It is recommended to revise the discussion, conclusion, and Table 3. | We thank the Reviewer for catching this. The Table 3 sample size error has been corrected: the Generation X subgroup, previously misreported, is now correctly shown as n = 65 and excluded from the age invariance analysis for the reasons described above. The Discussion and Conclusions have been revised accordingly to reflect age invariance as established for Generations Z and Y only, rather than across all age groups. |
