# Supplementary material for: Polish adaptation and validation of the sustainable career scale
Source: PLoS One. 2026 Sep 8;21(9):e0357496. doi: 10.1371/journal.pone.0357496 (PMC13552746; doi:10.1371/journal.pone.0357496)
Supplement: Peer review history

# Peer review: Decision Letter 0

**DOI:** 10.1371/journal.pone.0357496.r001

**Authors:** Rogis Baker

12 May 2026

Dear Dr. Grobelny,

Thank you for submitting your manuscript to PLOS ONE. After careful consideration, we feel that it has merit but does not fully meet PLOS ONE’s publication criteria as it currently stands. Therefore, we invite you to submit a revised version of the manuscript that addresses the points raised during the review process.

Please submit your revised manuscript by Jun 26 2026 11:59PM. If you will need more time than this to complete your revisions, please reply to this message or contact the journal office at plosone@plos.org. When you're ready to submit your revision, log on to https://www.editorialmanager.com/pone/ and select the 'Submissions Needing Revision' folder to locate your manuscript file.

- A letter that responds to each point raised by the academic editor and reviewer(s). You should upload this letter as a separate file labeled 'Response to Reviewers'.
- A marked-up copy of your manuscript that highlights changes made to the original version. You should upload this as a separate file labeled 'Revised Manuscript with Track Changes'.
- An unmarked version of your revised paper without tracked changes. You should upload this as a separate file labeled 'Manuscript'.

If you would like to make changes to your financial disclosure, please include your updated statement in your cover letter. Guidelines for resubmitting your figure files are available below the reviewer comments at the end of this letter.

If applicable, we recommend that you deposit your laboratory protocols in protocols.io to enhance the reproducibility of your results. Protocols.io assigns your protocol its own identifier (DOI) so that it can be cited independently in the future. For instructions see: https://journals.plos.org/plosone/s/submission-guidelines#loc-laboratory-protocols. Additionally, PLOS ONE offers an option for publishing peer-reviewed Lab Protocol articles, which describe protocols hosted on protocols.io. Read more information on sharing protocols at https://plos.org/protocols?utm_medium=editorial-email&utm_source=authorletters&utm_campaign=protocols.

As the corresponding author, your ORCID iD is verified in the submission system and will appear in the published article. PLOS supports the use of ORCID, and we encourage all coauthors to register for an ORCID iD and use it as well. Please encourage your coauthors to verify their ORCID iD within the submission system before final acceptance, as unverified ORCID iDs will not appear in the published article. *Only*  the individual author can complete the verification step; PLOS staff *cannot*  verify ORCID iDs on behalf of authors.

We look forward to receiving your revised manuscript.

Kind regards,

Rogis Baker, Ph.D

Academic Editor

PLOS One

**Journal Requirements:**

1. When submitting your revision, we need you to address these additional requirements.

Please ensure that your manuscript meets PLOS ONE's style requirements, including those for file naming. The PLOS ONE style templates can be found at

https://journals.plos.org/plosone/s/file?id=wjVg/PLOSOne_formatting_sample_main_body.pdf and

https://journals.plos.org/plosone/s/file?id=ba62/PLOSOne_formatting_sample_title_authors_affiliations.pdf

2. Please include your full ethics statement in the ‘Methods’ section of your manuscript file. In your statement, please include the full name of the IRB or ethics committee who approved or waived your study, as well as whether or not you obtained informed written or verbal consent. If consent was waived for your study, please include this information in your statement as well.

3. Please ensure that you refer to Figure in your text, if accepted, production will need this reference to link the reader to the figure.

4. If the reviewer comments include a recommendation to cite specific previously published works, please review and evaluate these publications to determine whether they are relevant and should be cited. There is no requirement to cite these works unless the editor has indicated otherwise. 

[Note: HTML markup is below. Please do not edit.]

Reviewers' comments:

**Comments to the Author**

1. Is the manuscript technically sound, and do the data support the conclusions?

Reviewer #1: No

Reviewer #2: Yes

**********

2. Has the statistical analysis been performed appropriately and rigorously?

Reviewer #1: No

Reviewer #2: Yes

**********

3. Have the authors made all data underlying the findings in their manuscript fully available?

The PLOS Data policy

Reviewer #1: No

Reviewer #2: Yes

**********

4. Is the manuscript presented in an intelligible fashion and written in standard English?

Reviewer #1: Yes

Reviewer #2: Yes

**********

**Reviewer #1:**  Thank you for the opportunity to review this manuscript, entitled 'Cross-Cultural Validation of the Sustainable Career Scale in the Polish Context'. The study addresses a relevant topic in career psychology and makes a potentially useful contribution by adapting and validating the Sustainable Career Scale in the Polish context. The multi-stage design, including qualitative assessment, pilot testing, confirmatory factor analysis, validity testing, and follow-up data, is appropriate in principle for a cross-cultural validation study.

However, in its current form, the manuscript requires substantial revision before it can be considered technically sound for publication. My main concerns relate to the clarity of the study contribution, the independence of the samples, the measurement invariance analysis, statistical reporting, interpretation of the hierarchical model, predictive validity claims, ethics reporting, and the strength of the conclusions.

Major comments

1. The contribution of the study should be framed more cautiously.

The manuscript provides new evidence for the Polish adaptation of the Sustainable Career Scale, which is valuable. However, the original scale has already been developed and validated in previous research, including evidence on factor structure, reliability, validity, predictive validity, and gender invariance. Therefore, the present contribution should be described more specifically as a Polish cultural adaptation and validation study, rather than as broad evidence for the universal cross-cultural applicability of the scale.

2. The independence of Study 1 and Study 2 needs clarification.

A major concern is that Study 1 and Study 2 appear to report very similar recruitment numbers and overlapping data collection periods. For example, both studies appear to report that 654 individuals started the survey. The authors should clearly explain whether Study 1 and Study 2 used fully independent samples, partially overlapping samples, or different analyses from the same recruitment wave. If the samples overlap, the manuscript should not present them as independent validation studies.

3. The measurement invariance analysis should be revised.

The manuscript states that measurement invariance was examined across gender and age groups. However, the current reporting seems to rely mainly on separate CFA models for different groups rather than formal multi-group measurement invariance testing. A proper invariance analysis should test configural, metric, scalar, and, where appropriate, residual invariance, using established criteria such as changes in CFI, RMSEA, and SRMR. Without such testing, the claim that the scale is invariant across gender or age groups is not sufficiently supported.

4. Several statistical reporting issues require correction.

There are inconsistencies in the reported sample sizes. For example, the subgroup sample sizes in the invariance table do not appear to align clearly with the final sample size reported for Study 1. The reported age-group sample size for Generation X also appears problematic given the total sample size. In addition, the Bartlett test reported in the pilot study seems to include a degree of freedom value that may not correspond to a 12-item factor analysis. The authors should carefully re-check all statistical values, sample sizes, degrees of freedom, and table entries.

5. The claim that the hierarchical four-factor model is superior is not fully supported.

The manuscript argues in favour of the hierarchical four-factor model. However, the reported fit indices seem to suggest that the four correlated-factor model may fit the data better on some criteria. The authors should provide a clearer comparison of competing models, including fit indices and, where appropriate, AIC, BIC, and chi-square difference tests. The justification for using a total SCS score should also be reconsidered and discussed more cautiously.

6. The EFA reporting in the pilot study is insufficient.

The exploratory factor analysis is reported rather briefly. The authors should provide a fuller factor loading matrix, including cross-loadings, eigenvalues, factor correlations, and communalities. Reporting only selected or target-factor loadings does not allow readers to evaluate the factorial clarity of the adapted scale.

7. The interpretation of AVE and discriminant validity needs clarification.

The manuscript appears to use AVE, the square root of AVE, and 'squared AVE' inconsistently. The authors should clarify exactly what is reported in the relevant tables and ensure that the Fornell-Larcker criterion is applied correctly. If the diagonal values in the correlation tables are AVE values rather than square roots of AVE, the interpretation should be revised.

8. The predictive validity evidence should be interpreted more cautiously.

Study 2 is presented as evidence of predictive validity. However, the follow-up sample is relatively small, and the analyses do not appear to control for baseline levels of the outcome variables. Therefore, the findings may be better described as lagged associations rather than strong evidence of predictive validity. If regression assumptions such as homoscedasticity or normality of residuals are not fully met, the authors should conduct appropriate robustness checks or soften the conclusions.

9. The ethics statement is internally inconsistent.

The submission information states that formal ethics approval was not required, whereas the manuscript states that approval was obtained from the ethics committee of Adam Mickiewicz University. This inconsistency must be resolved. The authors should clearly state whether the study received formal ethics approval, was granted an exemption, or did not require review under institutional rules. If approval was obtained, the name of the ethics committee, approval number, and approval date should be provided. The authors should also clarify how email addresses collected for the follow-up study were stored, separated from survey responses, and deleted or anonymised.

10. The conclusions should be more closely aligned with the evidence.

The findings support the reliability and validity of the Polish version of the SCS in the present samples. However, they do not justify broad claims about universal cross-cultural applicability across diverse cultural settings. The conclusion should be revised to state more cautiously that the study provides preliminary evidence for the Polish adaptation of the SCS in Polish worker samples, and that further research with independent, representative, and occupationally diverse samples is needed.

Minor comments

1. The terminology should be standardised throughout the manuscript. In particular, the manuscript appears to alternate between 'social empowerment' and 'social impact'. One term should be selected and used consistently.

2. The use of GPT-3.5 in the translation process should be described more transparently. The authors should clarify its exact role, how the AI-generated output was checked by human experts, and how conceptual equivalence was ensured.

3. The focus group component is underreported. More detail is needed on the participants, coding procedure, interpretation of responses, and how the qualitative findings informed item revision.

4. The manuscript contains several grammatical errors and repeated sentences. Careful language editing is recommended.

5. Some table labels should be checked. For example, if 'alpha when dropped' reports negative values or changes in alpha, the table heading should be revised to avoid misunderstanding.

6. The scoring of negatively worded health items should be explained more clearly, including whether reverse coding was applied before reliability, CFA, and validity analyses.

7. The authors should specify exactly what is available on OSF, including anonymised raw data, processed data, codebook, Polish scale items, scoring instructions, and analysis scripts.

8. The preregistration statement is too brief. The authors should clarify which analyses were preregistered and which analyses, if any, were exploratory.

Overall, the manuscript has potential, but substantial revision is required. The authors should clarify the sample structure, correct the statistical reporting errors, conduct formal measurement invariance testing, resolve the ethics inconsistency, and moderate the conclusions before the manuscript can be properly evaluated for publication.

**Reviewer #2:**  Thank you for giving me the opportunity to review this manuscript.

The overall research design is relatively rigorous, with well-established and appropriate statistical methods, clear empirical logic, and reasonable and orderly argumentation. It has certain theoretical and practical research value in the field of occupational psychology. However, there are also several issues that can be discussed, such as:

1.The content of lines 7-13 on page 7 is repetitive, and it is recommended to revise it.

2.In all three samples (pilot, Study 1, Study 2), the proportion of females exceeds 70%, the proportion of white-collar workers/service industry employees exceeds 70%, and the proportion of individuals with higher education exceeds 70%. It is evident that there are certain limitations in the representativeness of the samples. It is recommended to point this out more explicitly in the "Limitations" section.

3.The sample size of Generation X is below the minimum recommended sample size for CFA, leading to poor model fit. The author acknowledges this limitation but still concludes that "the data support H2". Additionally, is there an error in the sample size presented in Table 3? It is recommended to revise the discussion, conclusion, and Table 3.

**********

what does this mean?). If published, this will include your full peer review and any attached files.

If you choose “no”, your identity will remain anonymous but your review may still be made public.

**Do you want your identity to be public for this peer review?**  For information about this choice, including consent withdrawal, please see our Privacy Policy

Reviewer #1: No

Reviewer #2: No

**********

[NOTE: If reviewer comments were submitted as an attachment file, they will be attached to this email and accessible via the submission site. Please log into your account, locate the manuscript record, and check for the action link "View Attachments". If this link does not appear, there are no attachment files.]

To ensure your figures meet our technical requirements, please review our figure guidelines: https://journals.plos.org/plosone/s/figures

You may also use PLOS’s free figure tool, NAAS, to help you prepare publication quality figures: https://journals.plos.org/plosone/s/figures#loc-tools-for-figure-preparation.

NAAS will assess whether your figures meet our technical requirements by comparing each figure against our figure specifications.

# Author comment: Author response to Decision Letter 1

**DOI:** 10.1371/journal.pone.0357496.r002

30 Jun 2026

The detailed respond is attached

Attachment Submitted filename: 2025_SCS_main_PLoS_v5_rebuttal-letter.docx

# Peer review: Decision Letter 1

**DOI:** 10.1371/journal.pone.0357496.r003

**Authors:** Rogis Baker

23 Jul 2026

Dear Dr. Grobelny,

Thank you for submitting your manuscript to PLOS ONE. After careful consideration, we feel that it has merit but does not fully meet PLOS ONE’s publication criteria as it currently stands. Therefore, we invite you to submit a revised version of the manuscript that addresses the points raised during the review process.

Please submit your revised manuscript by Sep 06 2026 11:59PM. If you will need more time than this to complete your revisions, please reply to this message or contact the journal office at plosone@plos.org. When you're ready to submit your revision, log on to https://www.editorialmanager.com/pone/ and select the 'Submissions Needing Revision' folder to locate your manuscript file.

- A letter that responds to each point raised by the academic editor and reviewer(s). You should upload this letter as a separate file labeled 'Response to Reviewers'.
- A marked-up copy of your manuscript that highlights changes made to the original version. You should upload this as a separate file labeled 'Revised Manuscript with Track Changes'.
- An unmarked version of your revised paper without tracked changes. You should upload this as a separate file labeled 'Manuscript'.

If you would like to make changes to your financial disclosure, please include your updated statement in your cover letter. Guidelines for resubmitting your figure files are available below the reviewer comments at the end of this letter.

If applicable, we recommend that you deposit your laboratory protocols in protocols.io to enhance the reproducibility of your results. Protocols.io assigns your protocol its own identifier (DOI) so that it can be cited independently in the future. For instructions see: https://journals.plos.org/plosone/s/submission-guidelines#loc-laboratory-protocols. Additionally, PLOS ONE offers an option for publishing peer-reviewed Lab Protocol articles, which describe protocols hosted on protocols.io. Read more information on sharing protocols at https://plos.org/protocols?utm_medium=editorial-email&utm_source=authorletters&utm_campaign=protocols.

As the corresponding author, your ORCID iD is verified in the submission system and will appear in the published article. PLOS supports the use of ORCID, and we encourage all coauthors to register for an ORCID iD and use it as well. Please encourage your coauthors to verify their ORCID iD within the submission system before final acceptance, as unverified ORCID iDs will not appear in the published article. *Only*  the individual author can complete the verification step; PLOS staff *cannot*  verify ORCID iDs on behalf of authors.

We look forward to receiving your revised manuscript.

Kind regards,

Rogis Baker, Ph.D

Academic Editor

PLOS One

Journal Requirements:

If the reviewer comments include a recommendation to cite specific previously published works, please review and evaluate these publications to determine whether they are relevant and should be cited. There is no requirement to cite these works unless the editor has indicated otherwise.

Please review your reference list to ensure that it is complete and correct. If you have cited papers that have been retracted, please include the rationale for doing so in the manuscript text, or remove these references and replace them with relevant current references. Any changes to the reference list should be mentioned in the rebuttal letter that accompanies your revised manuscript. If you need to cite a retracted article, indicate the article’s retracted status in the References list and also include a citation and full reference for the retraction notice.

[Note: HTML markup is below. Please do not edit.]

Reviewers' comments:

Reviewer's Responses to Questions

**Comments to the Author**

Reviewer #1: (No Response)

Reviewer #2: All comments have been addressed

**********

2. Is the manuscript technically sound, and do the data support the conclusions?

Reviewer #1: Yes

Reviewer #2: (No Response)

**********

3. Has the statistical analysis been performed appropriately and rigorously?

Reviewer #1: Yes

Reviewer #2: Yes

**********

4. Have the authors made all data underlying the findings in their manuscript fully available?

The PLOS Data policy

Reviewer #1: Yes

Reviewer #2: Yes

**********

5. Is the manuscript presented in an intelligible fashion and written in standard English?

Reviewer #1: Yes

Reviewer #2: Yes

**********

Reviewer #1: Thank you for the careful and substantial revision. The manuscript has improved considerably, and the authors have adequately addressed most of my previous concerns. In particular, the revised manuscript now includes formal multigroup measurement-invariance analyses, clearer comparisons between the correlated four-factor and hierarchical models, more cautious interpretation of the prospective findings, additional robustness analyses, and a more appropriately qualified conclusion.

The manuscript is now close to being suitable for publication. However, several remaining issues should be clarified or corrected before acceptance.

1. Ethics approval chronology

The most important remaining issue concerns the ethics approval date. The manuscript reports that the data were collected between March and August 2024, whereas the stated ethical approval was issued on 17 March 2026.

Please clarify whether “2026” is a typographical error. If so, please provide the correct date and revise it consistently in the manuscript and submission information. If the approval was genuinely issued in 2026, please explain what prospective ethical approval, exemption, or institutional authorization covered the participant recruitment and data collection conducted in 2024.

2. Interpretation of the total SCS score

The revised analyses indicate that the four correlated-factor model fits better than the hierarchical model. Therefore, the interpretation of the total SCS score should be further qualified.

Please avoid implying that parsimony alone provides sufficient psychometric justification for the total score. The manuscript should state more clearly that the four subscale scores may be preferable when examining dimension-specific relationships, whereas the total score should be interpreted cautiously as a broad summary measure.

3. Convergent and discriminant validity

Please moderate the claims concerning the convergent and discriminant validity of the Health dimension. The relatively low square root of AVE, particularly in Study 2, should be acknowledged as a limitation.

The manuscript should also clarify how AVE was calculated in Study 2 and explain the limitations of applying the Fornell–Larcker criterion when the external constructs were not all assessed within a common latent-variable measurement model.

4. Study 2 reporting

Please correct or clarify the following points:

- The power analysis refers to four predictors, whereas the regression table appears to include the SCS and four demographic control variables.

- Statistical power should be reported as 1 − β = .95 rather than β = .95.

- Please report the actual follow-up interval, because the stated data-collection dates suggest that it may not have been exactly seven weeks for every participant.

- Please provide brief statistical details supporting the statement that the follow-up participants did not differ significantly from the initial sample.

- Please clarify that Pearson correlation primarily reflects rank-order stability. Reporting an ICC with a confidence interval would provide a more complete assessment of test–retest reliability, if feasible.

5. Cross-cultural claims

The revised Conclusions are appropriately cautious, but some statements in the Abstract remain too strong, particularly the claims of “robust empirical evidence” and validation “across cultural contexts.”

The findings would be more accurately described as providing preliminary evidence supporting the Polish adaptation of the SCS in the present samples. The authors may also consider revising the title to emphasize “Polish Adaptation and Validation.”

6. Sample independence

The response letter states that Study 1 and Study 2 used independent samples with no participant overlap. Please include this clarification explicitly in the manuscript and briefly explain how duplicate participation across the two studies was prevented or assessed.

7. Data availability and presentation

Please clarify whether the analysis scripts are publicly available in addition to the raw data, processed data, codebook, Polish scale, and scoring instructions.

A final consistency and language check is also needed. In particular:

- Clarify whether the second set of factor loadings in Table 1 represents EFA or CFA results.

- Add the full reference for Robinson (2017), if retained.

- Clarify whether the descriptive statistics for the negatively worded Health items were calculated before or after reverse scoring.

- Use the terms criterion, convergent, discriminant, and divergent validity consistently.

- Check the Polish wording of Item 10.

- Correct the remaining grammatical, punctuation, spacing, and formatting errors.

Subject to satisfactory clarification of the ethics approval chronology and correction of the points above, I believe the manuscript may be suitable for publication.

Recommendation: Minor Revision.

Reviewer #2: (No Response)

**********

what does this mean?). If published, this will include your full peer review and any attached files.

If you choose “no”, your identity will remain anonymous but your review may still be made public.

**Do you want your identity to be public for this peer review?**  For information about this choice, including consent withdrawal, please see our Privacy Policy

Reviewer #1: No

Reviewer #2: No

**********

[NOTE: If reviewer comments were submitted as an attachment file, they will be attached to this email and accessible via the submission site. Please log into your account, locate the manuscript record, and check for the action link "View Attachments". If this link does not appear, there are no attachment files.]

To ensure your figures meet our technical requirements, please review our figure guidelines: https://journals.plos.org/plosone/s/figures

You may also use PLOS’s free figure tool, NAAS, to help you prepare publication quality figures: https://journals.plos.org/plosone/s/figures#loc-tools-for-figure-preparation.

NAAS will assess whether your figures meet our technical requirements by comparing each figure against our figure specifications.

# Author comment: Author response to Decision Letter 2

**DOI:** 10.1371/journal.pone.0357496.r004

29 Jul 2026

We thank the Reviewer for the careful reading of our manuscript and for the constructive comments provided throughout the review process. We have addressed each of the remaining points below, indicating where changes have been made in the manuscript. We are grateful for the attention this review has given to the accuracy and clarity of our reporting, and we hope that the revised manuscript now meets the criteria for publication.

Attachment Submitted filename: 2025_SCS_main_PLoS_v6_rebuttal-letter.docx

# Peer review: Decision Letter 2

**DOI:** 10.1371/journal.pone.0357496.r005

**Authors:** Rogis Baker

18 Aug 2026

Polish Adaptation and Validation of the Sustainable Career Scale

PONE-D-25-68801R2

Dear Grobelny,

We’re pleased to inform you that your manuscript has been judged scientifically suitable for publication and will be formally accepted for publication once it meets all outstanding technical requirements.

Within one week, you’ll receive an e-mail detailing the required amendments. When these have been addressed, you’ll receive a formal acceptance letter and your manuscript will be scheduled for publication.

An invoice will be generated when your article is formally accepted. Please note, if your institution has a publishing partnership with PLOS and your article meets the relevant criteria, all or part of your publication costs will be covered. Please make sure your user information is up-to-date by logging into Editorial Manager at Editorial Manager® and clicking the ‘Update My Information' link at the top of the page. For questions related to billing, please contact billing support.

If your institution or institutions have a press office, please notify them about your upcoming paper to help maximize its impact. If they’ll be preparing press materials, please inform our press team as soon as possible -- no later than 48 hours after receiving the formal acceptance. Your manuscript will remain under strict press embargo until 2 pm Eastern Time on the date of publication. For more information, please contact onepress@plos.org.

Kind regards,

Rogis Baker, Ph.D

Academic Editor

PLOS One

Additional Editor Comments (optional):

Reviewers' comments:

Reviewer's Responses to Questions

**Comments to the Author**

Reviewer #1: All comments have been addressed

Reviewer #3: All comments have been addressed

**********

2. Is the manuscript technically sound, and do the data support the conclusions?

Reviewer #1: Yes

Reviewer #3: (No Response)

**********

3. Has the statistical analysis been performed appropriately and rigorously?

Reviewer #1: Yes

Reviewer #3: (No Response)

**********

4. Have the authors made all data underlying the findings in their manuscript fully available?

The PLOS Data policy

Reviewer #1: Yes

Reviewer #3: (No Response)

**********

5. Is the manuscript presented in an intelligible fashion and written in standard English?

Reviewer #1: Yes

Reviewer #3: (No Response)

**********

Reviewer #1: Thank you for submitting the revised version of the manuscript entitled “Polish Adaptation and Validation of the Sustainable Career Scale”.

I have carefully reviewed the revised manuscript and the authors’ responses to the previous reviewer comments. The authors have adequately addressed the concerns raised in the previous round of review.

The revised manuscript has been improved in terms of methodological clarity, reporting of statistical analyses, interpretation of findings, and data availability. The authors have provided sufficient information regarding the validation process, analytical procedures, and supporting materials.

I have no further major concerns regarding the manuscript. The study is technically sound, the conclusions are supported by the data, and the manuscript is presented clearly.

Therefore, I recommend acceptance of the manuscript for publication in PLOS ONE.

Reviewer #3: I thank the authors for their careful and comprehensive revision of the manuscript. I have reviewed the revised version as well as the point by point responses to my previous comments.

The authors have satisfactorily addressed all remaining concerns raised in the prior round of review. In particular, the chronology and nature of the ethical review are now clearly explained, and the corresponding information has been revised consistently throughout the manuscript. The interpretation of the total SCS score has also been appropriately qualified in light of the superior fit of the four correlated factor model.

The authors have further clarified the longitudinal interval and attrition analyses in Study 2, added an ICC to complement the test–retest correlation, moderated the claims regarding predictive validity and cross cultural generalizability, and explicitly acknowledged the limitations of the Health dimension. The revised title and Abstract more accurately reflect the scope of the study as a Polish adaptation and validation of the SCS.

Overall, I consider that the authors have responded fully and appropriately to my previous comments, and that the manuscript has been substantially improved. I have no further major concerns.

**********

what does this mean?). If published, this will include your full peer review and any attached files.

If you choose “no”, your identity will remain anonymous but your review may still be made public.

**Do you want your identity to be public for this peer review?**  For information about this choice, including consent withdrawal, please see our Privacy Policy

Reviewer #1: No

Reviewer #3: **Yes:**  Tsuneaki Kenzaka

**********

# Editor report: Acceptance letter

**DOI:** 10.1371/journal.pone.0357496.r006

**Authors:** Rogis Baker

PONE-D-25-68801R2

PLOS One

Dear Dr. Grobelny,

I'm pleased to inform you that your manuscript has been deemed suitable for publication in PLOS One. Congratulations! Your manuscript is now being handed over to our production team.

At this stage, our production department will prepare your paper for publication. This includes ensuring the following:

* All references, tables, and figures are properly cited

* All relevant supporting information is included in the manuscript submission,

* There are no issues that prevent the paper from being properly typeset

You will receive further instructions from the production team, including instructions on how to review your proof when it is ready. Please keep in mind that we are working through a large volume of accepted articles, so please give us a few days to review your paper and let you know the next and final steps.

Lastly, if your institution or institutions have a press office, please let them know about your upcoming paper now to help maximize its impact. If they'll be preparing press materials, please inform our press team within the next 48 hours. Your manuscript will remain under strict press embargo until 2 pm Eastern Time on the date of publication. For more information, please contact onepress@plos.org.

You will receive an invoice from PLOS for your publication fee after your manuscript has reached the completed accept phase. If you receive an email requesting payment before acceptance or for any other service, this may be a phishing scheme. Learn how to identify phishing emails and protect your accounts at https://explore.plos.org/phishing.

If we can help with anything else, please email us at customercare@plos.org.

Thank you for submitting your work to PLOS One and supporting open access.

Kind regards,

PLOS One Editorial Office Staff

on behalf of

Dr. Rogis Baker

Academic Editor

PLOS One
